# Neuromuscular interaction is required for neurotrophins-mediated locomotor recovery following treadmill training in rat spinal cord injury

**DOI:** 10.7717/peerj.2025

**Published:** 2016-05-11

**Authors:** Qinfeng Wu, Yana Cao, Chuanming Dong, Hongxing Wang, Qinghua Wang, Weifeng Tong, Xiangzhe Li, Chunlei Shan, Tong Wang

**Affiliations:** 1 Department of Rehabilitation Medicine, the First Affiliated Hospital of Nanjing Medical University, Nanjing, Jiangsu, China; 2 Department of Rehabilitation Medicine, affiliated Hospital of Nantong University, Nantong, China; 3 Jiangsu Province Hospital of TCM, Nanjing, Jiangsu, China; 4 Department of Anatomy and Neurobiology, Nantong University, Nantong, Jiangsu, China; 5 Laboratory Animal Center, Nantong University, Nantong, Jiangsu, China; 6 Research Center for Neurobiological, Xuzhou Medical Collage, Xuzhou, Jiangsu, China; 7 School of Rehabilitation Science, Shanghai University of Traditional Chinese Medicine, Shanghai, China

**Keywords:** Treadmill training, Spinal cord injury, Neurotrophins, Motor neurons, Neuromuscular activity, BDNF

## Abstract

Recent results have shown that exercise training promotes the recovery of injured rat distal spinal cords, but are still unclear about the function of skeletal muscle in this process. Herein, rats with incomplete thoracic (T10) spinal cord injuries (SCI) with a dual spinal lesion model were subjected to four weeks of treadmill training and then were treated with complete spinal transection at T8. We found that treadmill training allowed the retention of hind limb motor function after incomplete SCI, even with a heavy load after complete spinal transection. Moreover, treadmill training alleviated the secondary injury in distal lumbar spinal motor neurons, and enhanced BDNF/TrkB expression in the lumbar spinal cord. To discover the influence of skeletal muscle contractile activity on motor function and gene expression, we adopted botulinum toxin A (BTX-A) to block the neuromuscular activity of the rat gastrocnemius muscle. BTX-A treatment inhibited the effects of treadmill training on motor function and BDNF/TrKB expression. These results indicated that treadmill training through the skeletal muscle-motor nerve-spinal cord retrograde pathway regulated neuralplasticity in the mammalian central nervous system, which induced the expression of related neurotrophins and promoted motor function recovery.

## Introduction

Spinal cord injury (SCI) is a severe traumatic condition of the central nervous system (CNS), which leads to movement deficiency, sensory and autonomic nerve dysfunction, and severely influences the quality of life of the patient (Lu et al., 2012). Exercise training is one of the most widely used rehabilitation treatments in the clinic. Many studies have confirmed that exercise training can improve the recovery of locomotor function after SCI. Previous studies mainly focus on the changes at the SCI site per se, but the molecular and cellular process of the neuromuscular interaction by exercise training in spinal cord recovery has not been investigated yet.

Exercise training can induce morphological and molecular changes in the distal spinal cord after SCI. Petruska et al. (2007) found that exercise training can increase the number of nerve fibers in the white matter of the lumbar spinal cord after incomplete spinal cord injury (iSCI) in a rat model. Beaumont et al. (2004) found that exercise training can improve secondary neuroprotenial abnormality of distal motor neurons after SCI.

Moreover, Forssberg, Grillner & Halbertsma (1980) and Forssberg et al. (1980) found that treadmill training can restore a certain degree of walking ability after thoracic spinal cord transection in a cat model (Martinez & Rossignol, 2013). The distal spinal cord can be compensatory in controlling the movement of the lower limbs, particularly in the case of completely lost control of the CNS, which suggests that treadmill training can induce the functional recombination of the distal spinal cord after complete thoracic SCI to improve motor function of the lower limbs. Thus, the function of the distal spinal cord plays an important role in recovery of motor function after spinal cord transection. However, for incomplete SCI, some nerve fibers are reserved, and the function of remnant nerve fibers and the distal spinal cord might be involved in the recovery of motor function after SCI. In addition, the role of morphological and molecular changes of the distal spinal cord after SCI in the recovery process of motor function remains unclear.

In this study, we first treated rats with incomplete SCI at T10, then we carried out four weeks of treadmill training. Then, a complete spinal transection at T8 was performed. Morphological and molecular examinations revealed that exercise training benefited the recovery of injured spinal cords and increased the expression of neurotrophic factors. In addition, BTX-A, a neurotoxin that blocks the release of neurotransmitters at the neuromuscular junction, hindered the effects of treadmill training. Above all, neuromuscular activity enhances locomotor recovery in a dual spinal cord lesion model following treadmill training.

## Materials and Methods

### Spinal cord injury procedures

**Figure 1:**
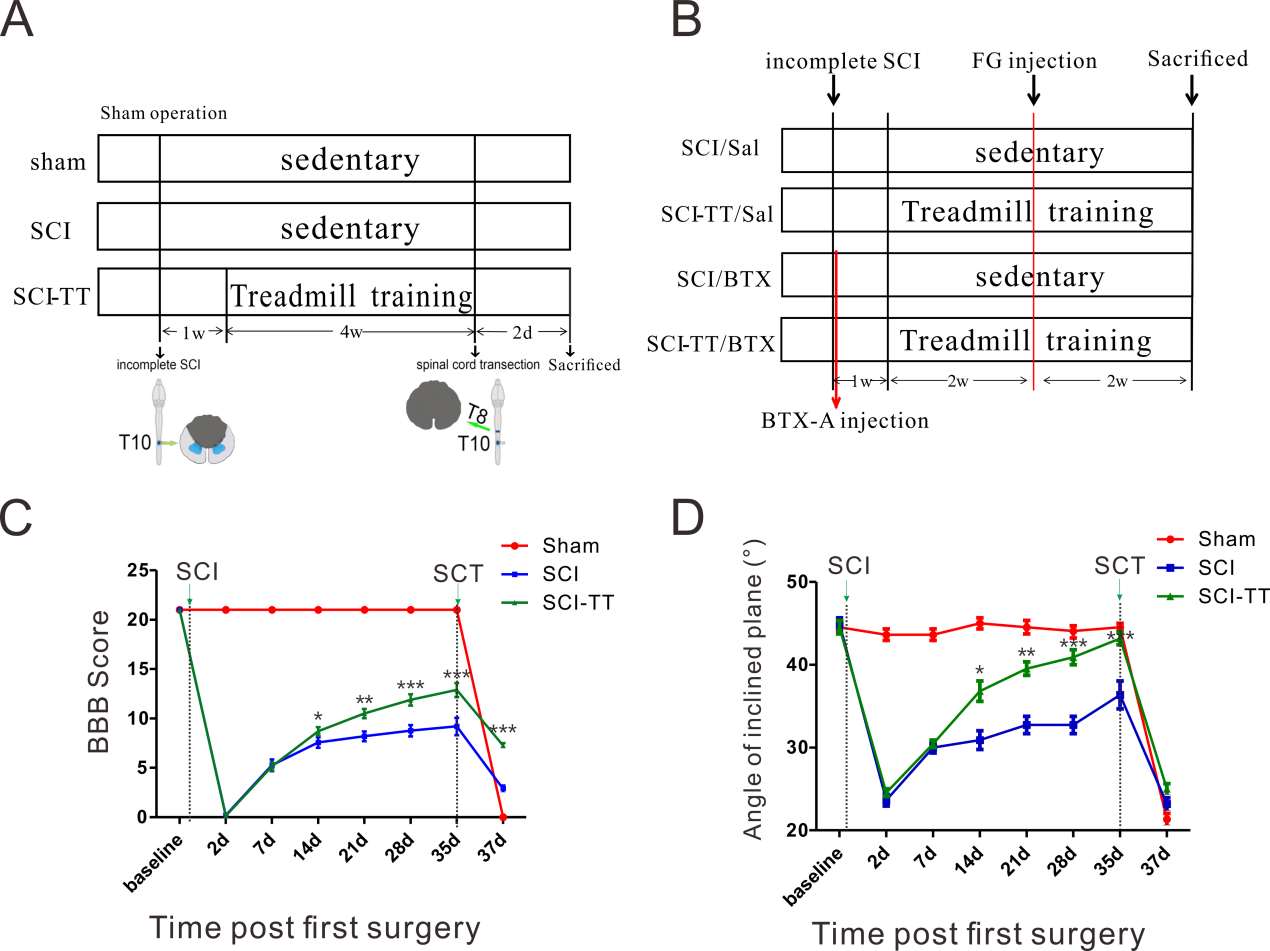
Experimental design and functional assessments. (A) The experimental procedures of the dual rat spinal lesion model and the time course in all rats are shown. T10: the 10th thoracic spinal cord, T8: the 8th thoracic spinal cord, TT: treadmill training. (B) The experimental procedures of the BTX-A injection and the time course in all rats are shown. FG: Fluoro Gold, TT: treadmill training, Sal: saline. (C) Treadmill-training significantly improved Basso, Beattie, and Bresnahan (BBB) scores starting from the second week up to the seventh week after SCI as compared to non-trained rats. Statistically significant differences were found among the three groups two days after spinal cord transection (SCT). (D) Treadmill-training also significantly increased the angle of the inclinedplane from the third week up to the seventh week after SCI as compared to non-trained rats. No statistically significant difference was found among the three groups two days after SCT. ^∗^*P* < 0.05, ^∗∗^*P* < 0.01, ^∗∗∗^*P* < 0.001.

Adult female Sprague-Dawley rats (SD, 220–250 g, Nanjing Medical University, *n* = 36) were randomly divided into three groups: Sham (*n* = 12), SCI (*n* = 12) and SCI + treadmill training (SCI-TT, *n* = 12). Rats were anesthetized via an intraperitoneal injection of 10% chloral hydrate (0.35 ml/kg). The spinous process and the vertebral lamina were removed to expose a circular region of dura at the T10 spinal level (Noble & Wrathall, 1985). By means of the New York University (NYU) Impactor System, an incomplete spinal cord injury was made by dropping a device rod (10 g) from a distance of 25 mm onto the intact exposed dura (Basso, Beattie & Bresnahan, 1996; Young, Kume-Kick & Constantini, 1994). Postoperative care included regular bladder expression and antibiotic treatment (Trimethoprim, 0.85 mg/kg) if bladder infections occurred. One week after surgery, rats were trained to walk on a customized treadmill for 4 weeks. At the end of the fifth week, the spinal cord was then completely transected at the T8 spinal level using surgical scissors. Two days later, all rats were anesthetized and perfused through the heart with 4% paraformaldehyde (Fig. 1A). The spinal cords were removed from the vertebral columns, post-fixed in the same fixative, and stored in 30% sucrose at 4 °C. Tissues were stored at - 70 °C until sectioned in a cryostat.

### BTX-A injection

Botulinum toxin type A (BTX-A) is a neurotoxin that blocks the release of neurotransmitters at the neuromuscular junction (Soljanik, 2013). Thus, BTX-A was used to paralyze the gastrocnemius muscle to determine the effects of neuromuscular interaction. Adult female SD rats (220–250 g, Nanjing Medical University, *n* = 48) were randomly divided into four groups: SCI/Saline (Sal) (*n* = 12), SCI-TT/Sal (*n* = 12), SCI/BTX (*n* = 12) and SCI-TT/BTX (*n* = 12). Rats were anesthetized via an intraperitoneal injection of 10% Hydrate hydrate (0.35 ml/kg). The SCI-TT/BTX group and SCI/BTX group were treated by injection on bilateral gastrocnemius muscle with BTX-A (Sigma, 1 ng/kg) (5 µl/ unilateral side; two sites/ unilateral side). The SCI/Sal group and SCI-TT/Sal group were given equal volumes of physiological saline (Fig. 1B). The incision sites were flushed liberally with a saline solution and the skin was sutured and disinfected. All rats were housed according to Animal care and all experiment procedures approved by the Animal Committee of the school (IACUC-13020121).

### Treadmill training

Rats were trained to walk on the treadmill one week after injury. The exercise schedule followed a regimen of 20 min per session, twice a day, five days per week, with a total training time period of four weeks. Treadmill speed began from 6.5 m/min and gradually increased to 11.5 m/min based on the rate of functional recovery.

### Fluoro Gold injection

In order to label the corresponding lumbar spinal cord motor neurons of the gastrocnemius muscle, a pressure injection of Fluoro-Gold (FG) in 2.5% in distilled water (100 nl) was made into the gastrocnemius 3 weeks after iSCI.

### Motor behavioral analysis

#### BBB (Basso-Beanie-Bresnahan) score

The 21 point BBB locomotion scale was used to assess locomotor recovery (Basso et al., 1996). The rats were placed in an open field (80 × 130 × 30 cm) and were observed individually for 5 min by two observers who were blinded to the allocation of the animals. The rats were tested in days 2, 7, 14, 21, 28, and 35 after the first surgery. Then, the rats were tested in day 2 after the second surgery.

#### Inclined plane task

Spinal cord injured mice were also tested using a second behavioral task, the inclined plane, which assesses an animal’s ability to maintain its position on a board which is raised in 5°increments and thus can be used as an index of hindlimb strength (Fehlings & Tator, 1992). The maximum angle at which a mouse is able to maintain its position for at least 5 s constitutes the inclined plane score. Mice were tested once a week throughout the survival period beginning at day 7 post-surgery.

### Nissl staining

For Nissl staining, spinal cord sections were stained with cresyl violet, dehydrated through graded alcohols (70, 95, 100% 2×), placed in xylene and coverslipped with DPX mountant. The morphology of the spinal cord was captured and Image-Pro Plus 6.0 (Media Cybernetics, Silver Spring, MD, USA) was used to perform quantitative analysis of motor neuron number and cross-sectional area in grey matter of spinal cord.

### Immunocytochemistry

The lumbar segments of the spinal cord (L3-L6) were frozen in Tissue-Tek optimal cutting temperature compound (O.C.T.) (VWR, Richmond, IL, USA) and 25 µm coronal sections were collected. Tissue sections were incubated in blocking solution consisting of 3% normal donkey serum (NDS; Jackson) and 0.3% Triton X-100 (Sigma-Aldrich, St. Louis, MO, USA) in PBS at room temperature (RT) for 30 min and then reacted with specific primary antibodies: NeuN (Millipore;1:500); c-fos (Sigma;1:200); BDNF (Abcam;1:50); TrkB (Millipore;1:100) at 4°C overnight. Each sample was washed with PBS three times, for 10 min each, and then stained by secondary antibodies for 1 h. Finally, cell nuclei were stained with DAPI. Images were acquired on a fluorescence microscope (Olympus BX 51; Olympus, Tokyo, Japan). Image-Pro Plus 6.0 (Media Cybernetics, Silver Spring, MD, USA) was used for quantitative analysis. During image acquisition, the illumination level of each imaging session was maintained by stabilizing the light source, and the settings of the camera and the lamp were constant.

### Western blotting

Protein samples were prepared from the lumbar segments of the spinal cord (L3-L6) of each group. Proteins were separated by 12% stacking SDS-PAGE and Western blotting was performed for NeuN (Millipore;1:1000); c-fos (Sigma;1:500); BDNF (Abcam;1:100); TrkB (Millipore;1:300) and *β* actin (1:2000; Cell Signaling Technology) was included as a loading control.

### Statistical analysis

All data were presented as mean ± SEM values, and were analyzed by one-way ANOVA and post-hoc Tukey’s tests for statistical significance between groups using SPSS Statistics 22.0 (IBM) software, with significance measured at *, *P* < 0.05; **, *P* < 0.01; or ***, *P* < 0.001.

## Results

### Treadmill training improves motor function recovery after dual spinal cord lesion

First, we applied the Basso-Beanie-Bresnahan (BBB) score to evaluate the time course of the motor alterations that were induced by the incomplete SCI or by the sham operation in the three experimental groups. BBB score testing showed rats undergone treadmill training exhibited significantly enhanced locomotor recovery which starts from the second week post-injury to the fifth week (Fig. 1C). An inclined plane test was also used to evaluate the effect of treadmill training on hind-limb muscle strength of rats after iSCI. It showed that the angle of the inclined plane was significantly higher in the SCI + treadmill training (SCI-TT) group as compared to the SCI group two weeks after occurrence of the lesion (Fig. 1D). In addition, two days after complete spinal transection, motor function was completely lost in the sham group, and only a few joints of the hind legs could be moved in the SCI group (2.64 ± 1.03). More importantly, the SCI-TT group retained good hind limb motor function, even with a heavy load burden (7.45 ± 0.82).

### Treadmill training alleviates secondary injury in lumbar spinal motor neurons after dual spinal cord lesion

**Figure 2:**
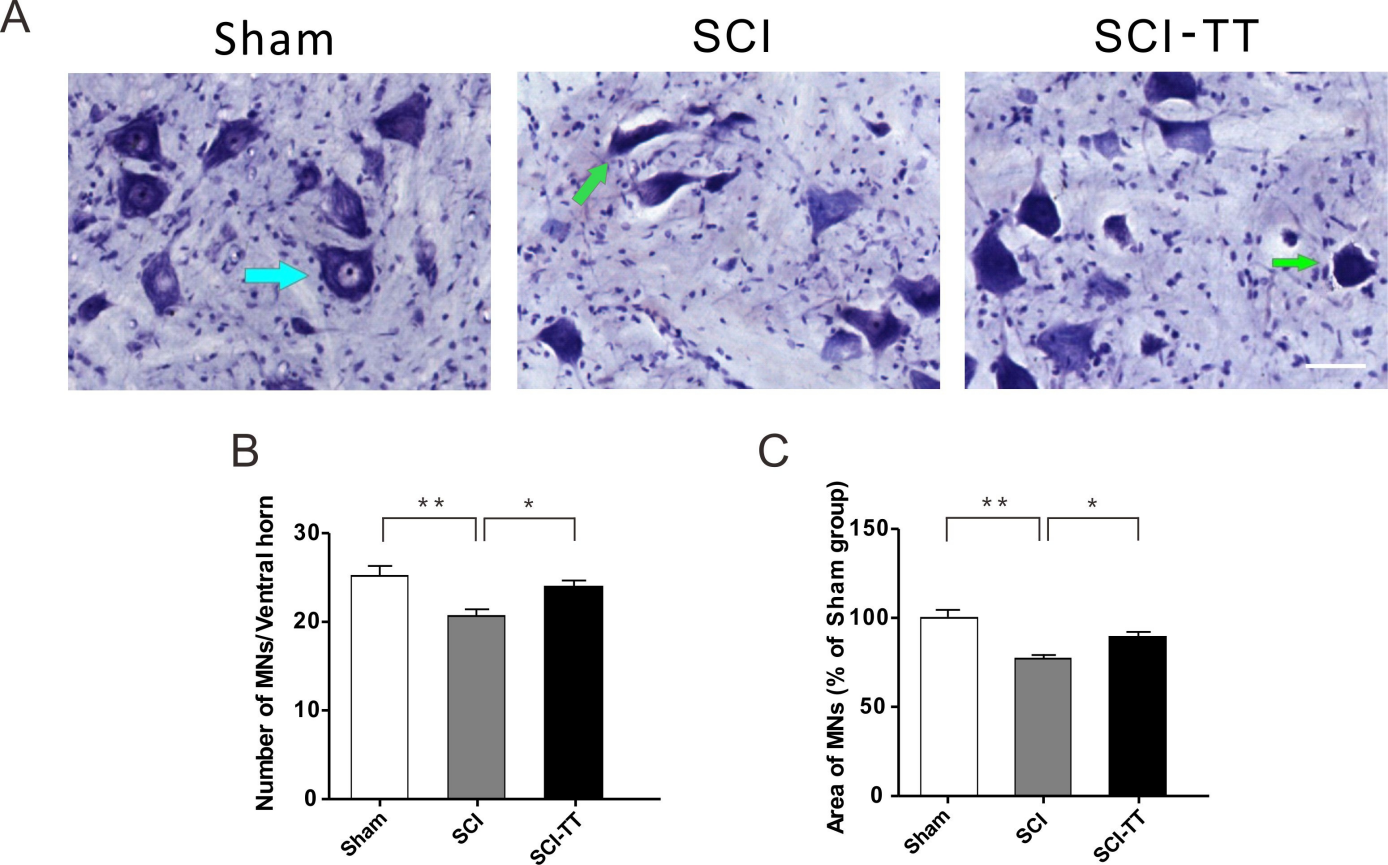
Treadmill training alleviates secondary injury of the lumbar spinal motor neurons after SCI. (A) Nissl staining of the lumbar spinal cord transverse section. The blue arrow indicates the normal motor neurons, and the green arrow indicates the abnormal motor neurons. Scale bars, 50 µm. (B) Shows the statistical graph of the mean number of ventral horn motor neurons among the three groups. (C) Shows the statistical graph of the relative area of motor neurons (% of sham group) among the three groups. ^∗^*P* < 0.05, ^∗∗^*P* < 0.01.

In the sham group, the motor neurons of the lumbar spinal cord’s ventral horn had a regular shape, clear nucleus, more neurites and more Nissl bodies in the cytoplasm. In the SCI group, part of the motor neurons had irregular shapes, deep colors and reduced volumes (Fig. 2A). The mean number of ventral horn motor neurons in the SCI-TT group was 23.97 ± 1.63 as compared to the SCI group 20.66 ± 1.92 (Fig. 2B). Furthermore, the cross-sectional area in the SCI-TT group was greater than that in the SCI group (i.e., 77.24 ± 1.91% in the SCI group and 89.42 ± 1.72% in the SCI-TT group relative to the sham group; Fig. 2C).

### Treadmill training enhances BDNF/TrkB expression in lumbar spinal cord after dual spinal cord lesion

**Figure 3:**
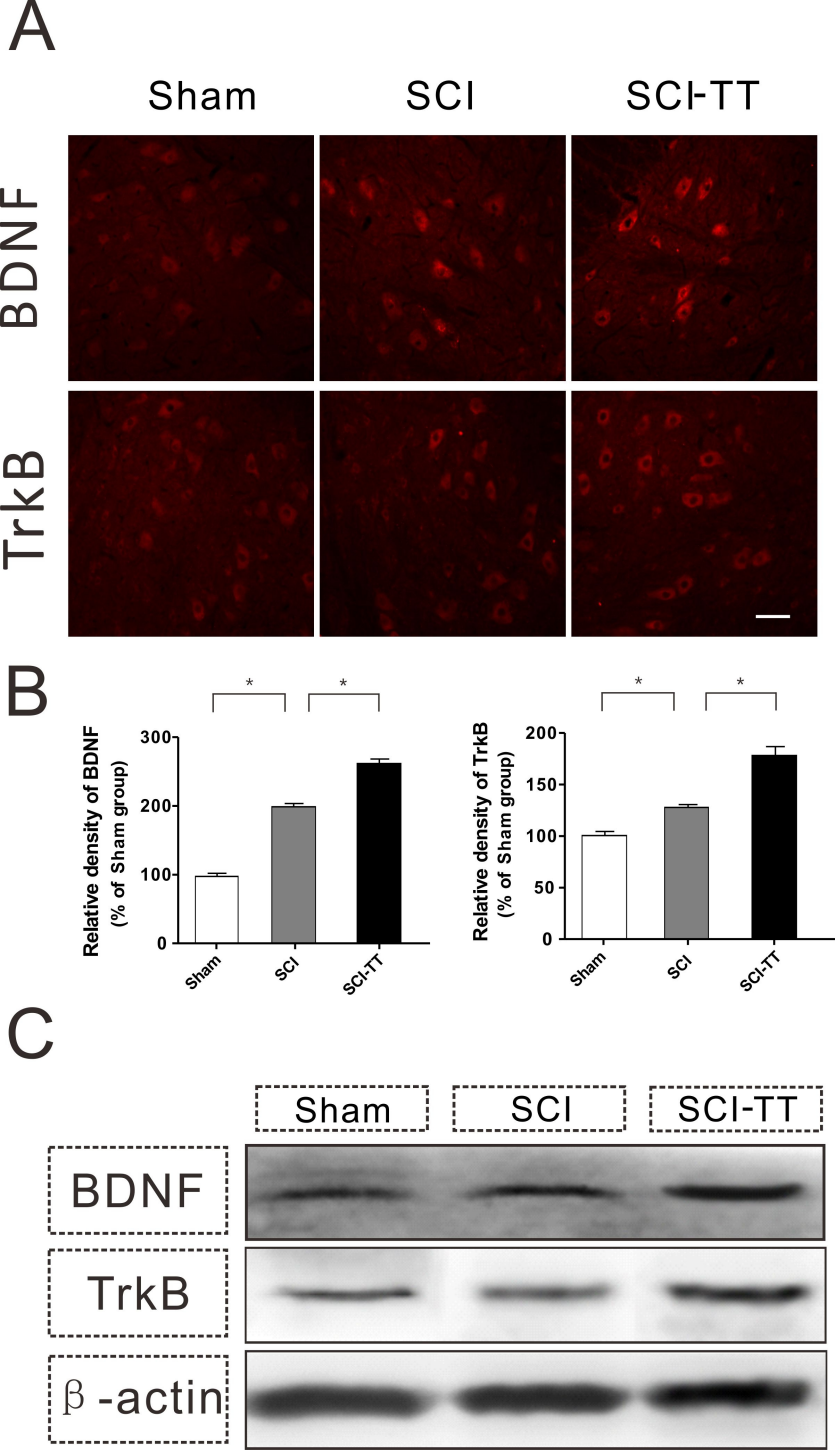
Treadmill training enhances the expression of both BDNF/TrkB in the lumbar spinal cord after SCI. (A) The expression of BDNF and TrkBin the lumbar spinal cord was determined by immunofluorescence staining among the three groups. Scale bars, 100 µm. (B) Shows the statistical graph of the relative density of BDNF and TrkB (% of sham group) among the three groups. ^∗^*P* < 0.05. (C) Western blotting analysis of BDNF and TrkB expression in the lumbar spinal cord among the three groups.

Histological analysis of the BDNF and TrkB expression in the distal lumbar spinal segments showed that both BDNF and TrkB fluorescence intensity in the SCI-TT group were higher than those in the SCI group (Figs. 3A and 3B). Additionally, the results of Western immunoblotting showed that treadmill training increased the expression levels of both BDNF and TrkB in the distal lumbar spinal cord after a dual spinal cord lesion (Fig. 3C).

### Improved motor function was inhibited by BTX-A intervention after treadmill training

**Figure 4:**
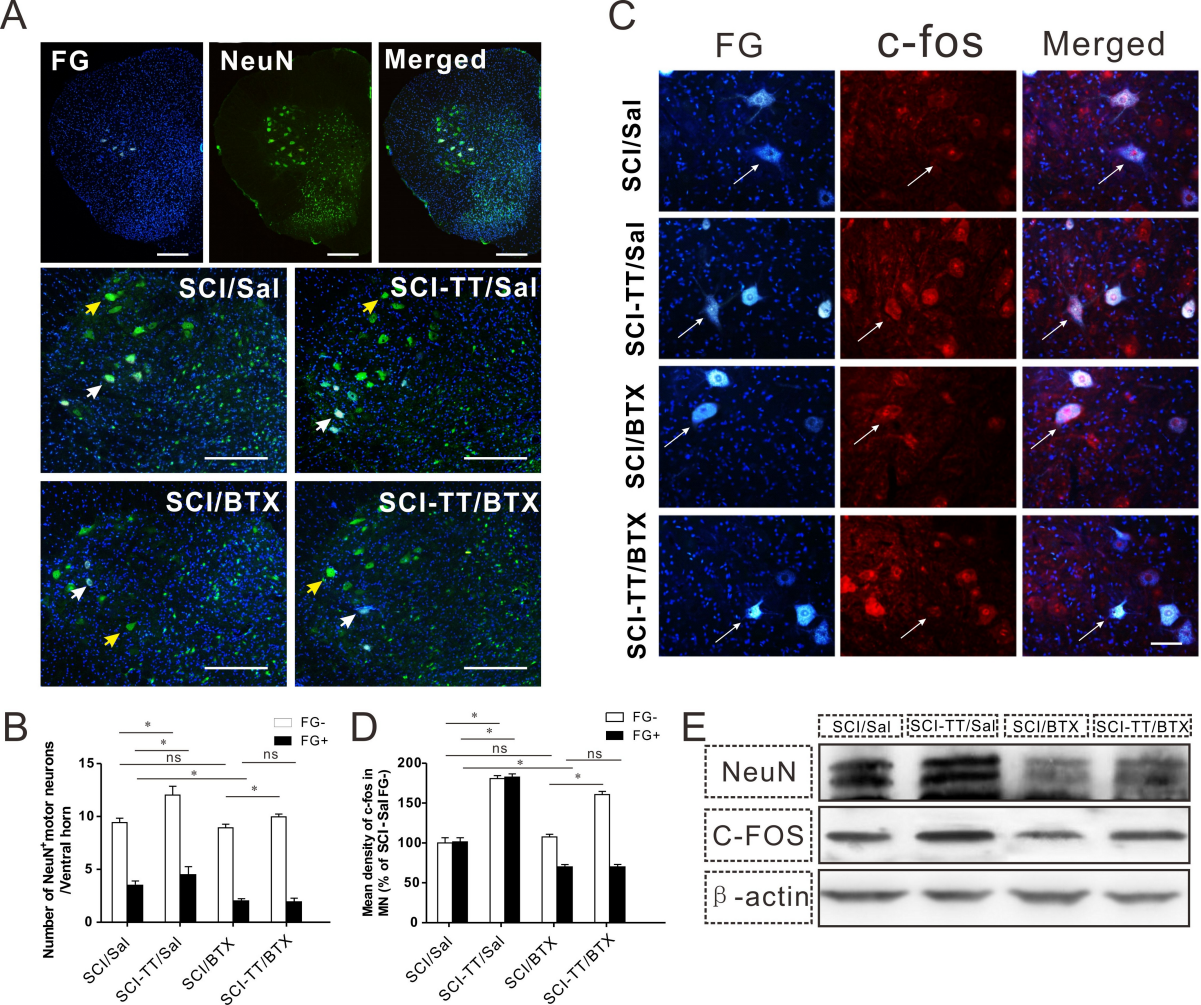
The improvement in motor function after treadmill training was inhibited with BTX-A injection. (A) The first row of the three images shown are of low magnification immunofluorescence staining; FG^+^ motor neurons (white), NeuN^+^ motor neurons (green), FG^+^/NeuN^+^ motor neurons (lightgreen). The second and the third row show high magnification immunofluorescence staining images of the four groups. The yellow arrow shows FG^-^/NeuN^+^ motor neurons, and the white arrow shows FG^+^/NeuN^+^ motor neurons. Scale bars, 500 µm. (B) Shows the statistical graph of the number of NeuN^+^ cells in the lumbar spinal cord of FG^+^ and FG^-^ motor neurons. (C) The expression of c-fos in the lumbar spinal cord FG^+^ and FG^-^ motor neurons was determined by immunofluorescence staining among the four groups. In addition, c-fos labeling (red) in FG-positive motor neurons (white) is shown. Overlap of c-fos and FG labeling (pink) indicates FG positive motor neurons that expressed c-fos. Scale bars, 50 µm. (D) Shows the statistical graph of the relative mean density of c-fos (% of SCI/Sal FG^-^). (E) Shows Western blot analysis of NeuN and c-fos expression in the lumbar spinal cord among the four groups. ^∗^*P* < 0.05.

The number of NeuN^+^ cells in the lumbar spinal cord FG^+^ and FG^-^ motor neurons was greater in the SCI-TT/Sal group than that in the SCI/Sal group (Figs. 4A and 4B), which was consistent with the change of fluorescence intensity of c-fos (Figs. 4C and 4D). The number of NeuN^+^ cells and the fluorescence intensity of c-fos in FG^-^ motor neurons was greater in the SCI-TT/BTX group than that in the SCI/BTX group. However, there was no significant difference in FG^+^ motor neurons (Figs. 4A–4D).

The expression of NeuN and c-fos in the lumbar spinal cord by Western blotting showed that the level of NeuN and c-fos was significantly higher in the SCI-TT/Sal group than that in the SCI/Sal group. Additionally, the levels of NeuN and c-fos were significantly higher in the SCI-TT/BTX group than those in the SCI/BTX group (Fig. 4E), which suggested that other lower limb muscles of the rat could still play a major role in movement, even after injection with BTX-A.

### BDNF and TrkB expression after treadmill training was inhibited after BTX-A injection

**Figure 5:**
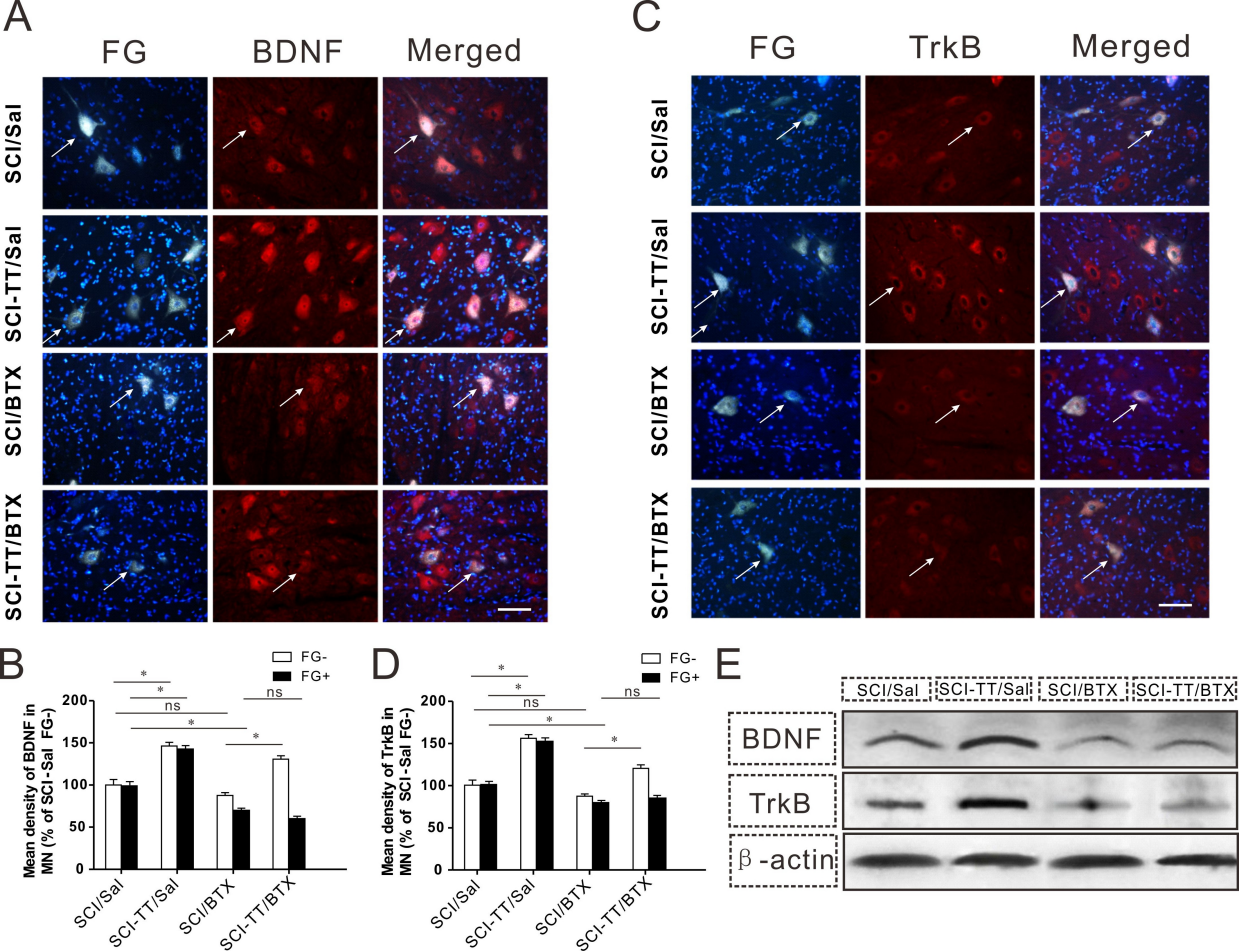
The expression of BDNF and TrkB after treadmill training was inhibited after BTX-A injection. (A) The expression of BDNF in the lumbar spinal cord FG^+^ and FG^-^ motor neurons was determined by immunofluorescence staining among the four groups. BDNF labeling (red) in FG-positive motor neurons (white) is also shown. The overlap of BDNF and FG labeling (pink) indicates FG positive motor neurons expressing BDNF. Scale bars, 50 µm. (B) Shows the statistical graph of the relative mean density of BDNF in the motor neurons (% of SCI/Sal FG^-^). (C) The expressionof TrkBin the lumbar spinal cord FG^+^ and FG^-^ motor neurons was determined by immunofluorescence staining among the four groups. TrkB labeling (red) in FG-positive motor neurons (white) is also shown. Overlap of TrkB and FG labeling (pink) indicates FG positive motor neurons expressing TrkB. Scale bars, 50 µm. (D) Shows the statistical graph of the relative mean density of TrkB in the motor neurons (% of SCI/Sal FG^-^). (E) Western immuno-blotting analysis of BDNF and TrkB expression in lumbar spinal cord among the four groups. ^∗^*P* < 0.05.

Histological analysis showed BDNF and TrkB fluorescence intensity in the lumbar spinal cord of FG^+^ and FG^-^ motor neurons was higher in the SCI-TT/Sal group than that in the SCI/Sal group. Furthermore, BDNF and TrkB fluorescence intensities in FG^-^ motor neurons were higher in the SCI-TT/BTX group than those in the SCI/BTX group, although there was no significant difference in FG^+^ motor neurons (Figs. 5A and 5D). Western blotting showed that the levels of BDNF and TrkB were significantly higher in the SCI-TT/Sal group than those in the SCI/Sal group. However, there was no significant difference between the SCI-TT/BTX group and the SCI/BTX group (Fig. 5E). This outcome suggested that the expression of BDNF and TrkB after iSCI was closely related to neuromuscular activity.

## Discussion

Previous studies have shown that exercise training is an effective way to promote the functional recovery from spinal cord injury (Oza & Giszter, 2015). However, the mechanism has remained unclear. Some studies have found that exercise training promotes the recovery from SCI by promoting axonal sprouting or by opening the bypass from the injured site of the traumatized spinal cord (Goldshmit et al., 2008), or by enhancing functional reconstruction in the cerebral cortex (Kao et al., 2011).

Firstly, the results of this study showed that the functional changes in the lumbar spinal cord play an important role in the recovery of SCI in rats after treadmill training based on the dual spinal lesion model. Secondly, BTX-A injection was used to reduce the neuromuscular activity of the gastrocnemius muscle, and we found that neuromuscular activity is required for locomotor recovery in SCI rats following treadmill training.

### The role of the lumbar spinal cord in the recovery of SCI following treadmill training

After complete spinal transection, the motor ability was completely lost in the Sham group, but certain movement functions were retained in the SCI and SCI-TT groups. In addition, the motor capability in the SCI-TT group was obviously superior to that found in the SCI group (Fig. 1). Complete spinal transection completely severed the corticospinal tract, and motor function preservation of the rat hind limbs was independent of the corticospinal tract and cerebral motor cortex. These results indicated that treadmill training promotes motor function rehabilitation of spinal cord injury by promoting spinal cord restructuring below the level of the incomplete lesion or by reducing secondary SCI. Our result is similar to the results reported by Barriere et al. (2008) using the dual spinal lesion model in the cat.

This study showed that the secondary injury might occur in lumbar spinal motor neurons, including motor neuron apoptosis, axonal loss, Nissl substance loss, and other factors beyond the scope of this discussion. The number of apoptotic motor neurons in the lumbar spinal cord decreased after treadmill training, which suggested that exercise training played a protective role in distal spinal motor neurons at the genetic level. However, the mechanisms of how to achieve this desirable protection is still not clear yet.

There are a series of changes in the spinal cord microenvironment after SCI. Among these beneficial factors are the functional expression of NGF (Brown, Ricci & Weaver, 2004), BDNF (Gomez-Pinilla et al., 2007), synaptophysin (Krassioukov & Weaver, 1996), growth-associated protein (gap-43) (Teng et al., 2002) and other factors. BDNF is an investigative hot-spot of current research, and plays an important role in promoting axonal growth, synaptic reconstruction, and alleviating secondary injury of the neuron in the spinal cord microenvironment (Ying et al., 2008). Our study showed that levels of BDNF and TrkB in the distal lumbar spinal cord in the dual spinal cord lesion model were higher than those in the sham group, similar to the results reported by Ying et al. (2005). We hypothesized that treadmill training might operate the protective effect over the distal spinal cord by increasing the gene expression of BDNF and TrkB in the spinal cord.

### Neuromuscular activity plays an important role in the recovery of SCI after treadmill training

It remains unclear precisely how treadmill training influences the distal spinal cord. Previous studies showed that exercise training could stimulate activity-dependent plasticity in the distal SCI through sensory feedback and in the remaining descending tracts (Edgerton et al., 2001; Thomas & Gorassini, 2005). Exercise training might influence the distal spinal cord through motor nerve retrograde transport. Wang et al. (2012) found that exercise training might retroactively change the threshold value of motor neurons and the peripheral nerve conduction ability in order to adapt to the decline of the ascending pathway.

In order to extrapolate the mechanism of exercise training promoting the reorganization of the distal spinal cord after SCI, the rat gastrocnemius muscle was injected with BTX-A to inhibit acetylcholine release from the motor endplate. At the same time, FG was injected into the gastrocnemius to mark corresponding motor neurons in the lumbar spinal cord (Hodgetts, Simmons & Plant, 2013). We found that the expression of BDNF and TrkB in the gastrocnemius muscle dominated the corresponding segmental spinal motor neurons, and that their expression decreased after injection with BTX-A.

Previous studies showed that the expression of BDNF depends on neuronal activity. BTX-A interrupts the protective effect of exercise training on motor neurons, which suggests that exercise training can induce the expression of BDNF in the spinal cord through a skeletal muscle-motor nerve reverse pathway. In addition, BTX-A can reduce the expression of BDNF in the spinal cord and muscle after SCI (Gomez-Pinilla et al., 2002). We speculate that BTX-A inhibits muscle contraction in the motor processes, thus decreases the expression of BDNF in the spinal cord and muscle after SCI, and consequently reduce the beneficial effect of exercise training on motor function.

Inhibiting muscle activity by injecting BTX-A to the rat gastrocnemius muscle and observing the impact on motor neurons was also studied. It is known that NeuN is a stable and sensitive neuron-specific marker (Wolf et al., 1996). In this experiment, we determined the function of motor neurons by detecting the expression of NeuN and counting the number of NeuN-positive cells. The results showed that the expression of NeuN increased in the lumbar cord of the rat by treadmill training after iSCI (Fig. 5E) and the number of motor neurons was higher than that found in untrained SCI rat model (Figs. 5A–5D). This finding suggests that treadmill training can reduce secondary apoptosis of motor neurons after SCI. However, the decrease in the number of motor neurons in the lumbar spinal cord was more obvious after BTX-A treatment, which suggested that maintenance of the function of motor neurons may depend on peripheral muscular activity. Secondary injury of motor neurons after SCI was probably due to a lack of muscle activity of the paralytic limbs, and this injury aggravated movement dysfunction. The results suggest that the contraction of muscle is essential in reducing secondary injury of motor neurons by treadmill training.

We also detected the expression of c-fos, which is a marker of the functional status of neurons and particularly of the lower motor neuron. C-fos is a proto-oncogene, and its expression is increased when neurons are excited (Zhang et al., 1992).We found that locomotor training can obviously increase c-fos expression in motor neurons, while c-fos expression was decreased after neuromuscular activity was inhibited by BTX-A. The increasing and decreasing trend of c-fos expression is accordingly consistent with the neuromuscular activity.

In addition to BDNF, many cytokines are expressed in skeletal muscle following exercise, such as IL-1*β*, IL-6, IL-8, IL-10, TNF-*α* and so on, some of which are neuroprotective (Peake et al., 2015). Whether these multiple cytokines play an important role in the recovery of SCI in rats after treadmill training based on our dual spinal lesion model is worth of being further studied.

In conclusion, treadmill training can increase BDNF and TrkB expression in the distal spinal cord after iSCI, prevent secondary injury of motor neurons in the lumbar cord, and promote the recovery of motor functions. In addition, neuromuscular activity is important in promoting iSCI functional recovery, and the skeletal muscle-motor nerve-spinal cord retrograde pathway plays an important role in the functional recovery from iSCI.

## Supplemental Information

10.7717/peerj.2025/supp-1 Data S1 Raw data-figure 1

10.7717/peerj.2025/supp-2 Data S2 Raw data-figure 2

10.7717/peerj.2025/supp-3 Data S3 Raw data-figure 3

10.7717/peerj.2025/supp-4 Data S4 Raw data-figure 4

10.7717/peerj.2025/supp-5 Data S5 Raw data-figure 5

## Additional Information and Declarations

### Data Availability

The following information was supplied regarding data availability: The raw data has been supplied as Supplemental Information.
