# Supplementary figures and images for: Neuromuscular interaction is required for neurotrophins-mediated locomotor recovery following treadmill training in rat spinal cord injury
Source: PeerJ. 2016 May 11;4:e2025. doi: 10.7717/peerj.2025 (PMC4867713; doi:10.7717/peerj.2025)

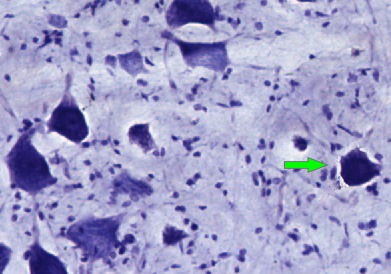

Supplement: Data S2 [file peerj-04-2025-s002.zip › raw data-figure 2/SCI-TT.jpg]

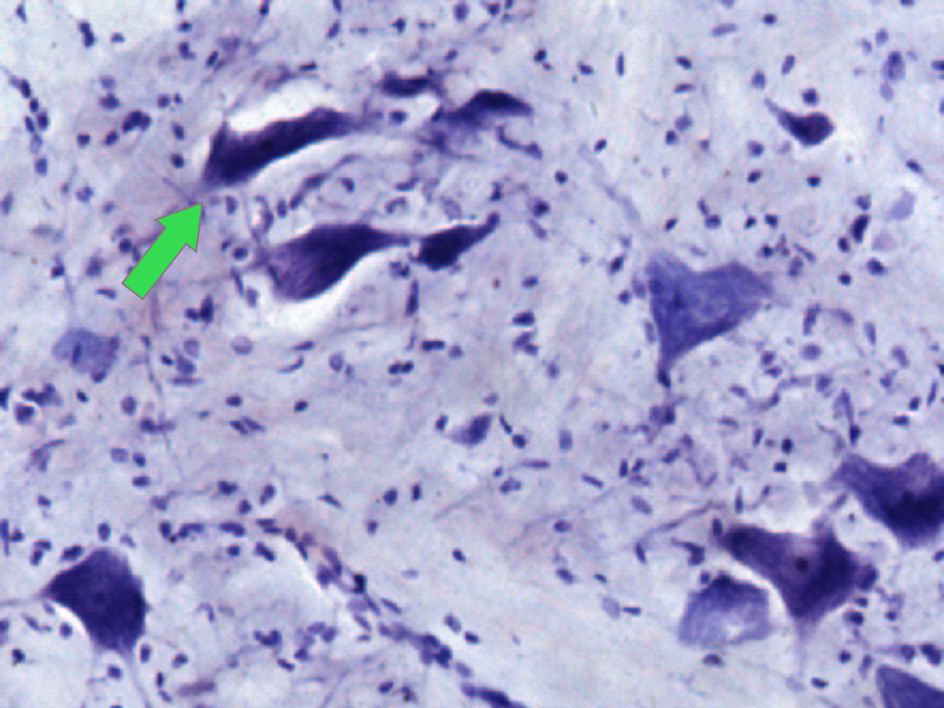

Supplement: Data S2 [file peerj-04-2025-s002.zip › raw data-figure 2/SCI.jpg]

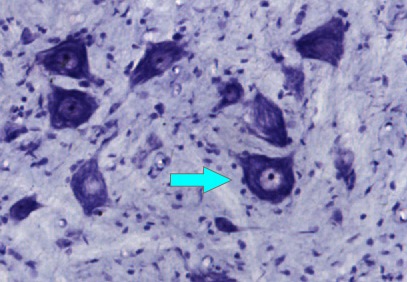

Supplement: Data S2 [file peerj-04-2025-s002.zip › raw data-figure 2/sham.jpg]

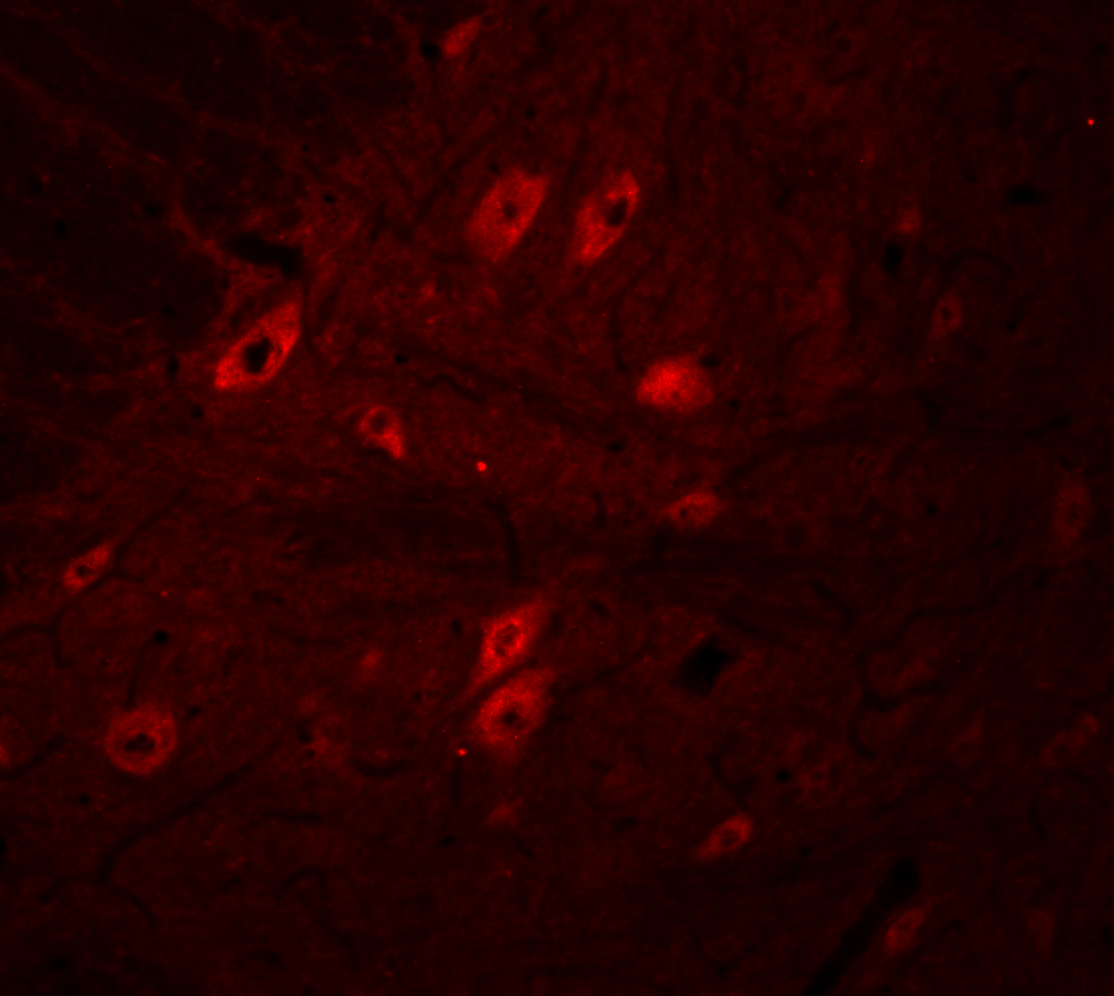

Supplement: Data S3 [file peerj-04-2025-s003.zip › raw data-figure 3/figure 3A/BDNF-SCI-TT.tif]

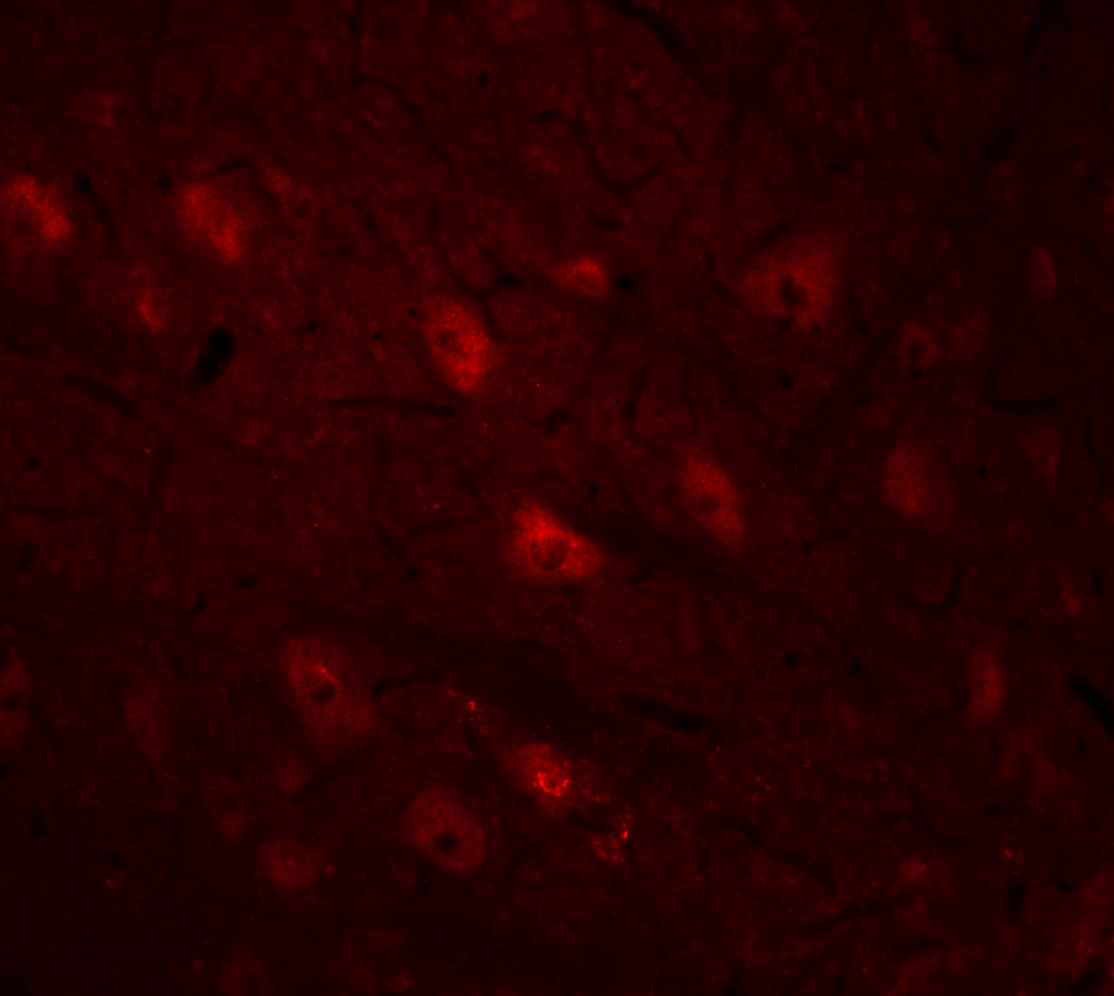

Supplement: Data S3 [file peerj-04-2025-s003.zip › raw data-figure 3/figure 3A/BDNF-SCI.tif]

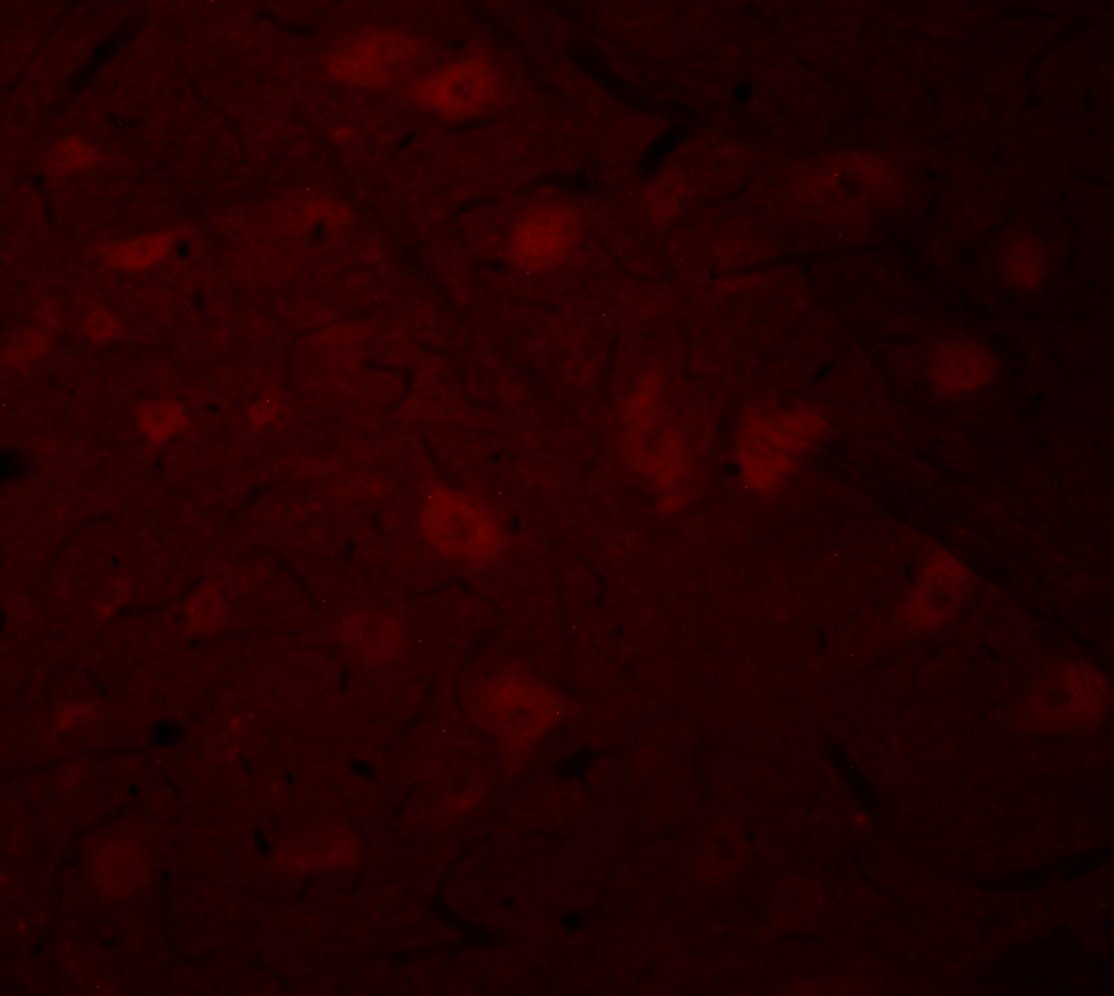

Supplement: Data S3 [file peerj-04-2025-s003.zip › raw data-figure 3/figure 3A/BDNF-sham.tif]

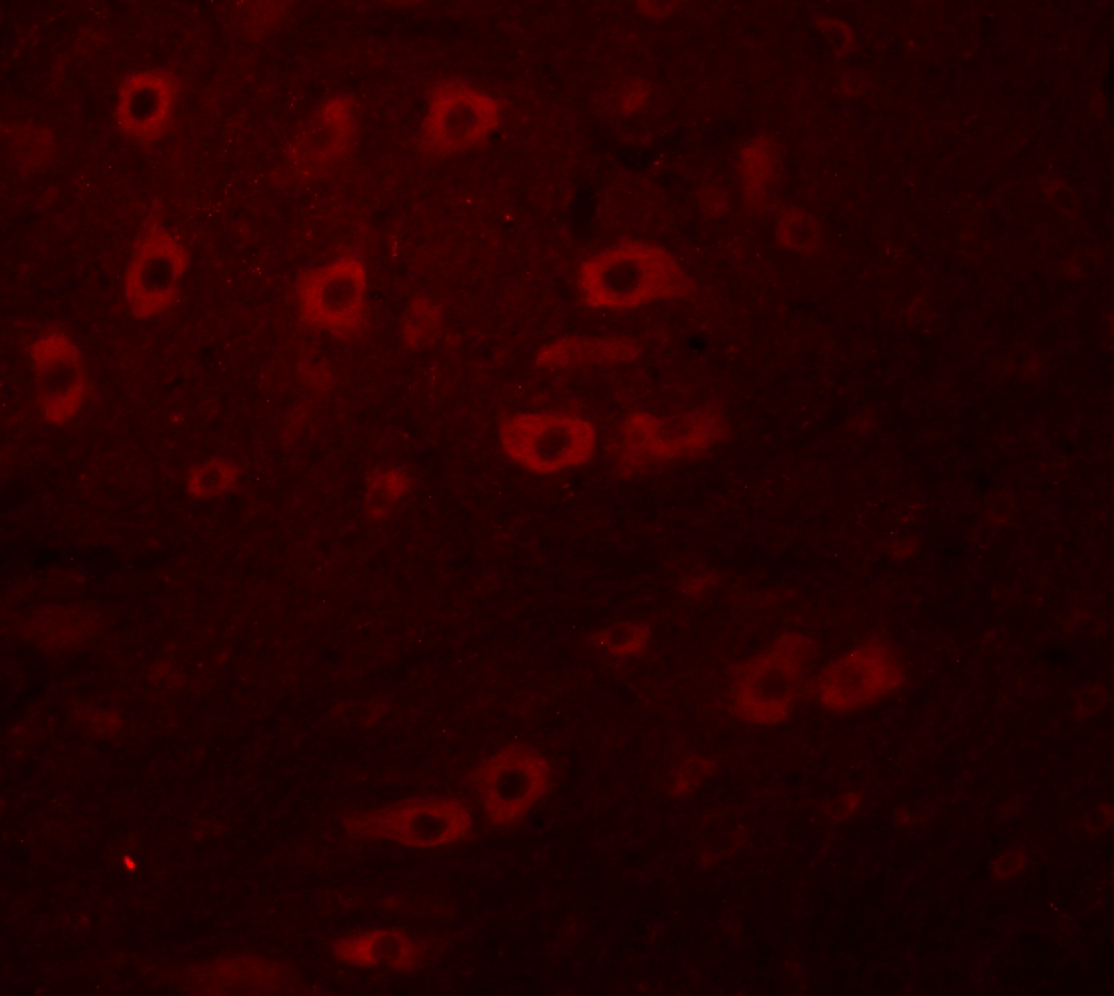

Supplement: Data S3 [file peerj-04-2025-s003.zip › raw data-figure 3/figure 3A/TrkB SCI TT.tif]

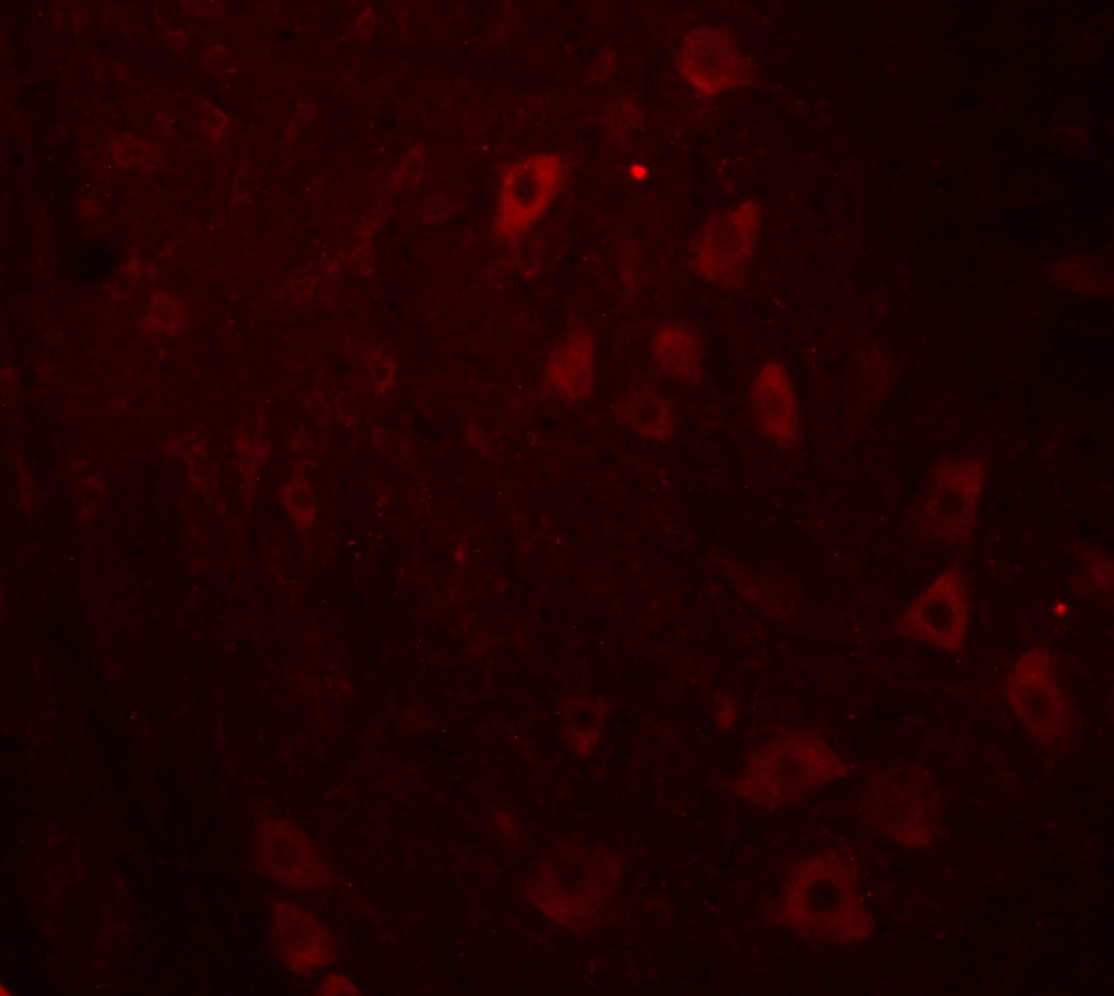

Supplement: Data S3 [file peerj-04-2025-s003.zip › raw data-figure 3/figure 3A/TrkB SCI.tif]

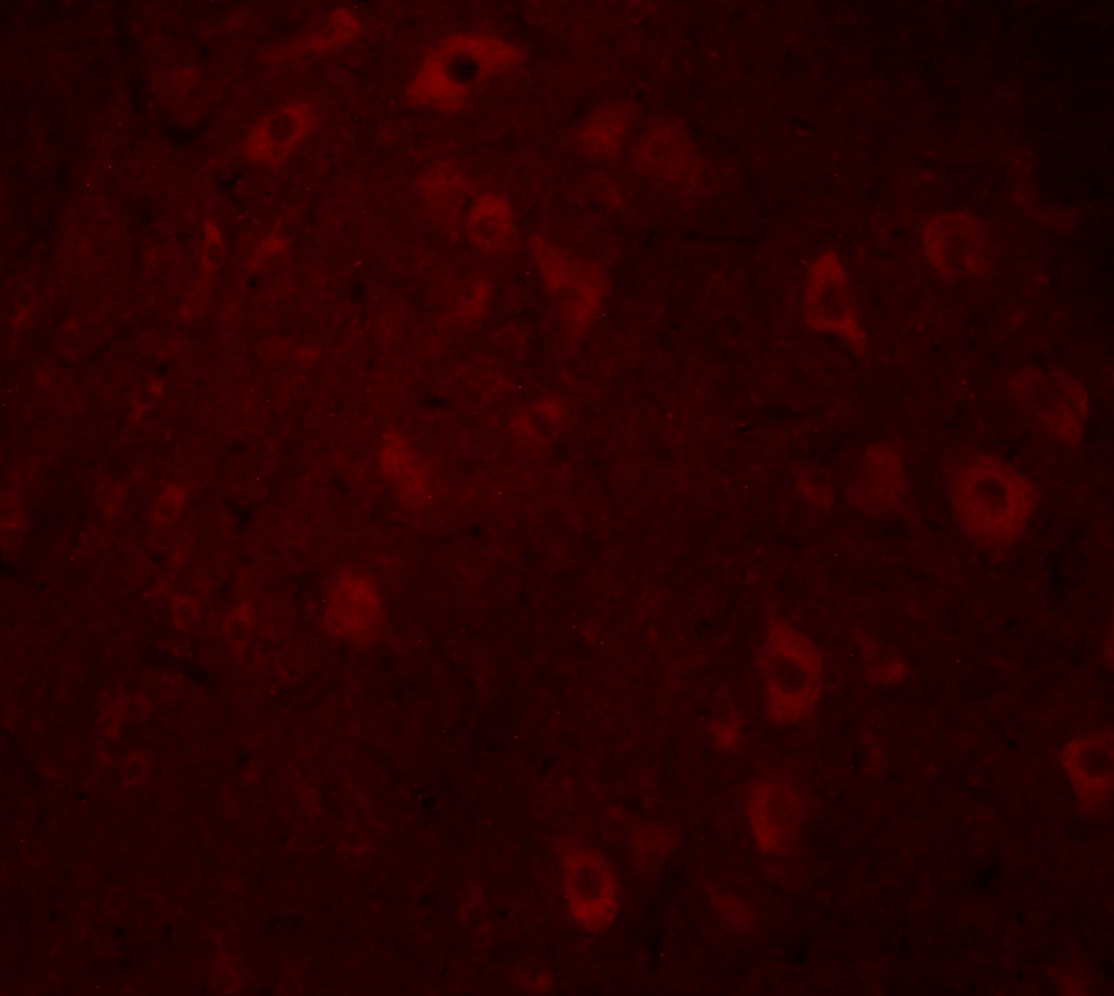

Supplement: Data S3 [file peerj-04-2025-s003.zip › raw data-figure 3/figure 3A/TrkB sham.tif]

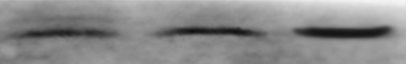

Supplement: Data S3 [file peerj-04-2025-s003.zip › raw data-figure 3/figure 3C/BDNF.tif]

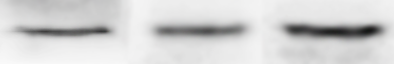

Supplement: Data S3 [file peerj-04-2025-s003.zip › raw data-figure 3/figure 3C/TrkB.tif]

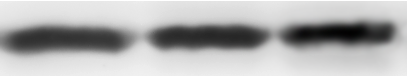

Supplement: Data S3 [file peerj-04-2025-s003.zip › raw data-figure 3/figure 3C/tublin.tif]

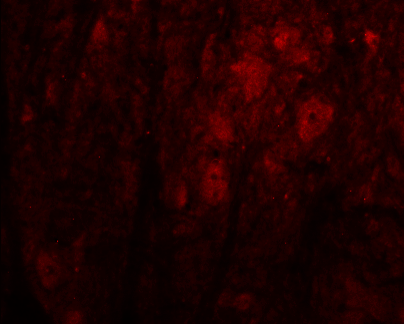

Supplement: Data S4 [file peerj-04-2025-s004.zip › raw data-figure 5/figure 5A/SCI-BTX-BDNF.tif]

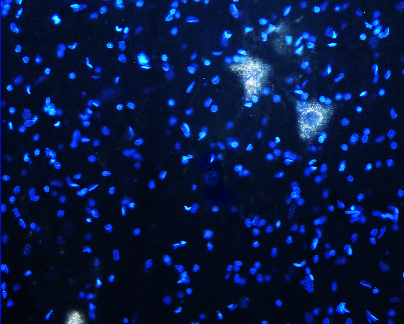

Supplement: Data S4 [file peerj-04-2025-s004.zip › raw data-figure 5/figure 5A/SCI-BTX-FG.tif]

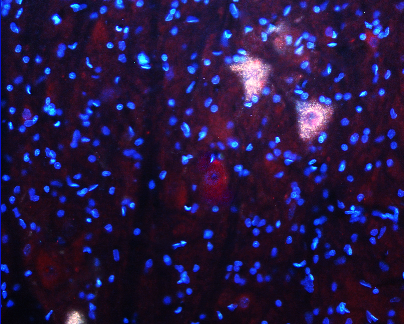

Supplement: Data S4 [file peerj-04-2025-s004.zip › raw data-figure 5/figure 5A/SCI-BTX-merged.tif]

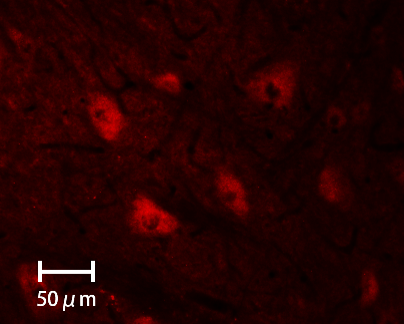

Supplement: Data S4 [file peerj-04-2025-s004.zip › raw data-figure 5/figure 5A/SCI-Sal-BDNF.tif]

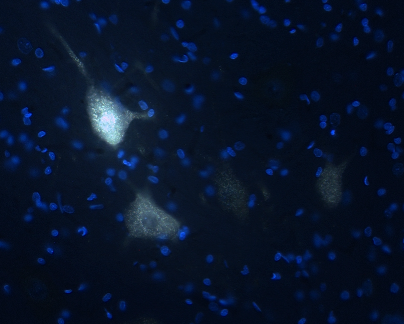

Supplement: Data S4 [file peerj-04-2025-s004.zip › raw data-figure 5/figure 5A/SCI-Sal-FG.tif]

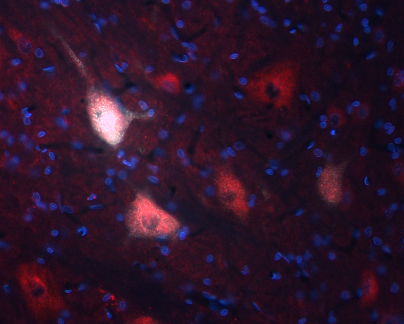

Supplement: Data S4 [file peerj-04-2025-s004.zip › raw data-figure 5/figure 5A/SCI-Sal-merged.tif]

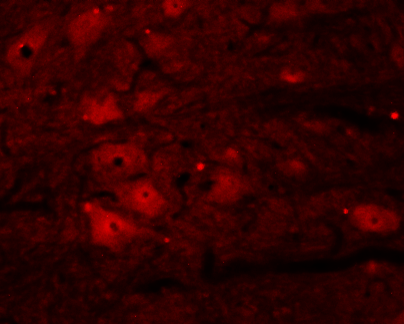

Supplement: Data S4 [file peerj-04-2025-s004.zip › raw data-figure 5/figure 5A/SCI-TT-BTX-BDNF.tif]

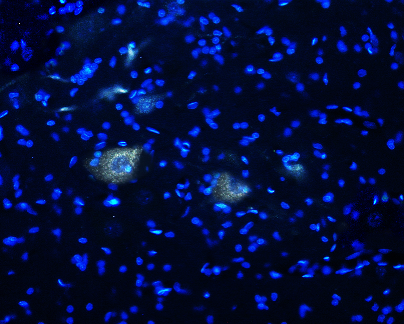

Supplement: Data S4 [file peerj-04-2025-s004.zip › raw data-figure 5/figure 5A/SCI-TT-BTX-FG.tif]

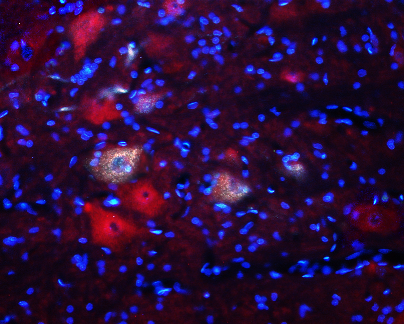

Supplement: Data S4 [file peerj-04-2025-s004.zip › raw data-figure 5/figure 5A/SCI-TT-BTX-merged.tif]

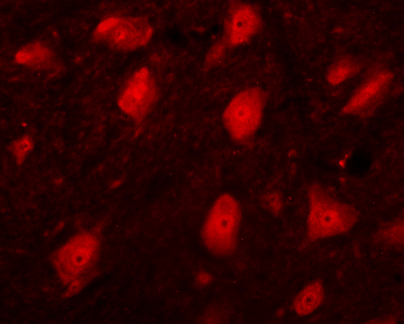

Supplement: Data S4 [file peerj-04-2025-s004.zip › raw data-figure 5/figure 5A/SCI-TT-Sal-BDNF.tif]

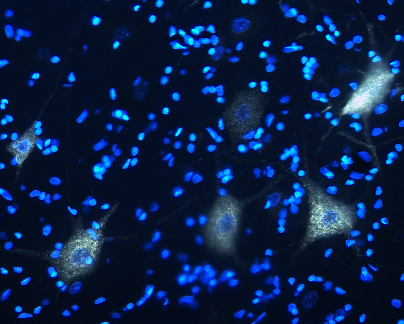

Supplement: Data S4 [file peerj-04-2025-s004.zip › raw data-figure 5/figure 5A/SCI-TT-Sal-FG.tif]

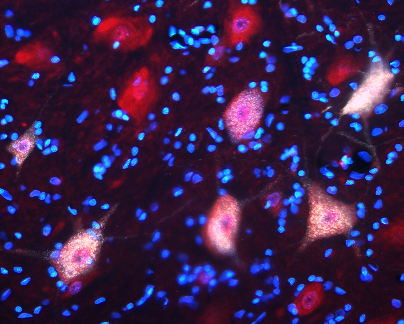

Supplement: Data S4 [file peerj-04-2025-s004.zip › raw data-figure 5/figure 5A/SCI-TT-Sal-meged.tif]

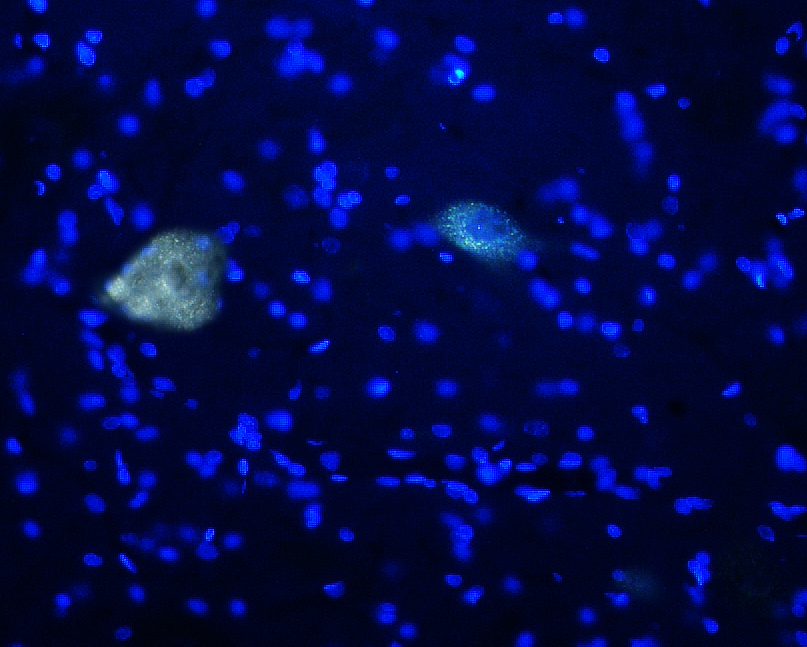

Supplement: Data S4 [file peerj-04-2025-s004.zip › raw data-figure 5/figure 5C/SCI-BTX-FG.tif]

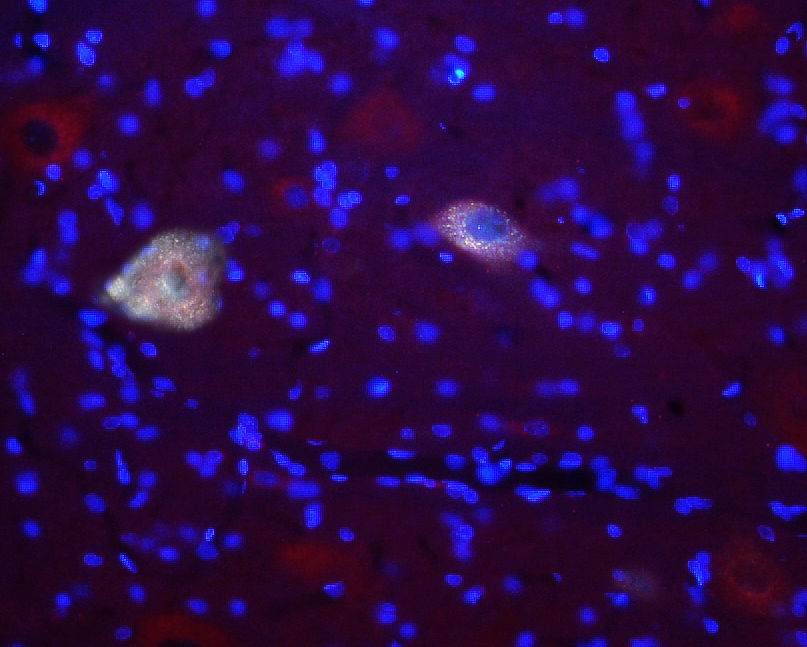

Supplement: Data S4 [file peerj-04-2025-s004.zip › raw data-figure 5/figure 5C/SCI-BTX-merged.tif]

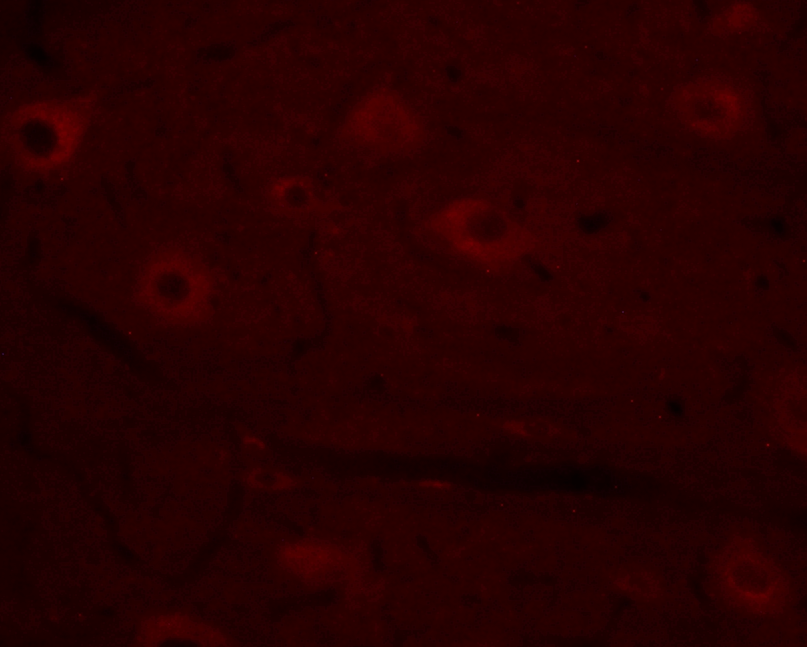

Supplement: Data S4 [file peerj-04-2025-s004.zip › raw data-figure 5/figure 5C/SCI-BTX-TrkB.tif]

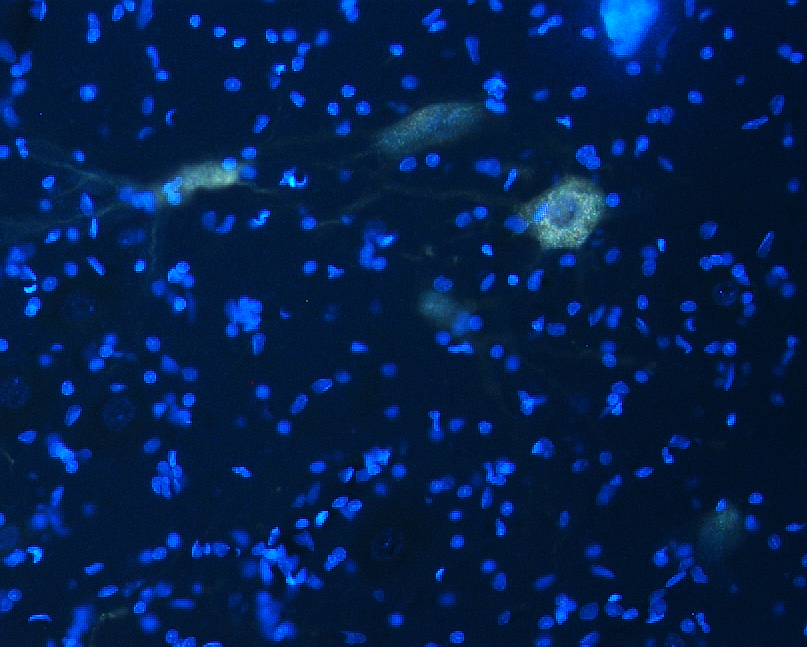

Supplement: Data S4 [file peerj-04-2025-s004.zip › raw data-figure 5/figure 5C/SCI-Sal-FG.tif]

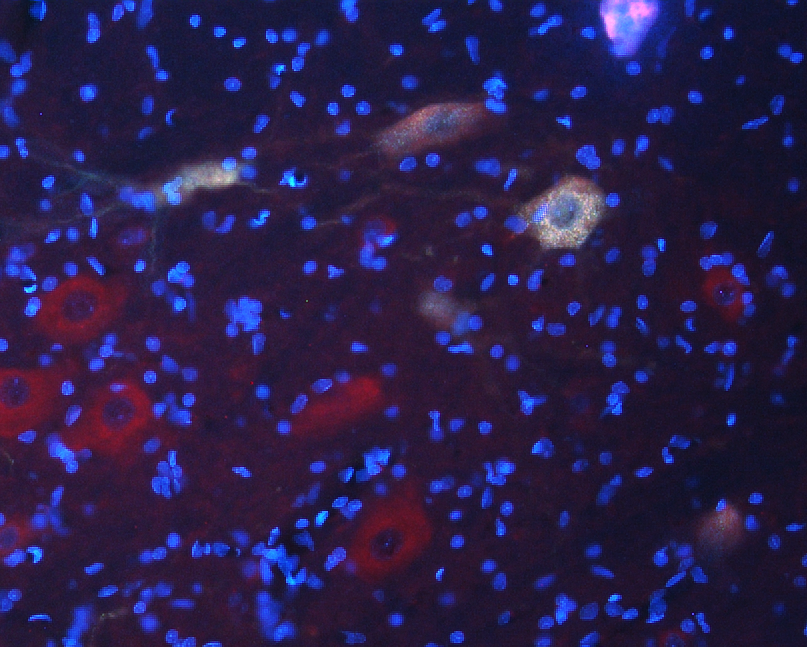

Supplement: Data S4 [file peerj-04-2025-s004.zip › raw data-figure 5/figure 5C/SCI-Sal-merged.tif]

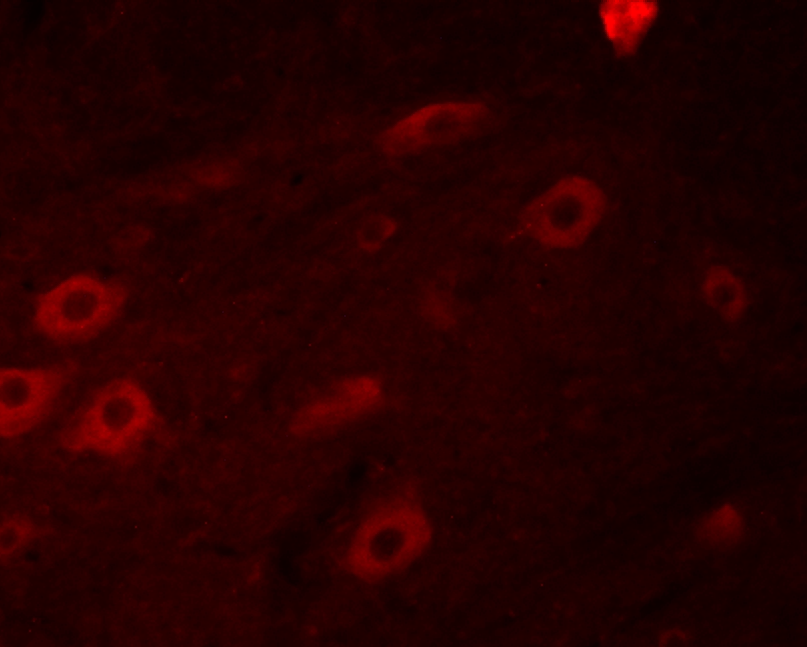

Supplement: Data S4 [file peerj-04-2025-s004.zip › raw data-figure 5/figure 5C/SCI-Sal-TrkB.tif]

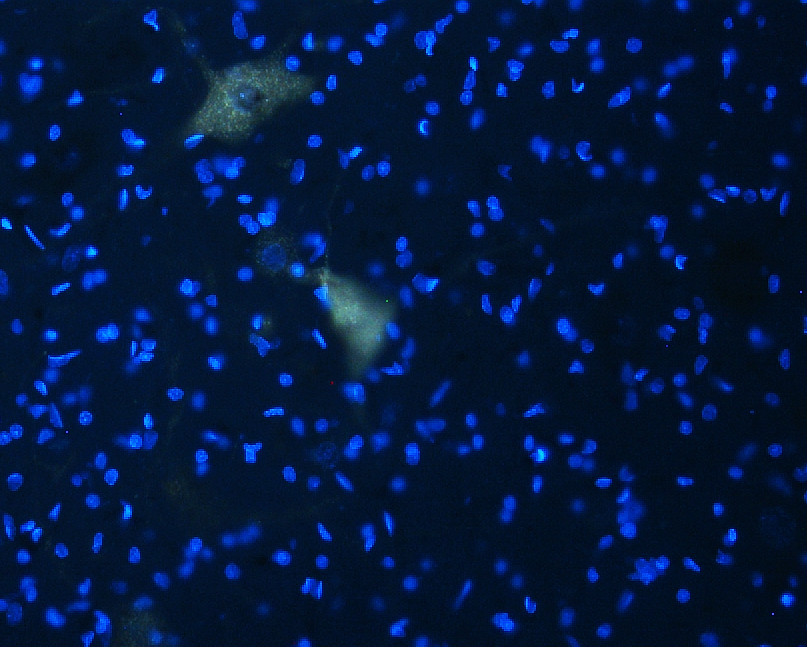

Supplement: Data S4 [file peerj-04-2025-s004.zip › raw data-figure 5/figure 5C/SCI-TT-BTX-FG.tif]

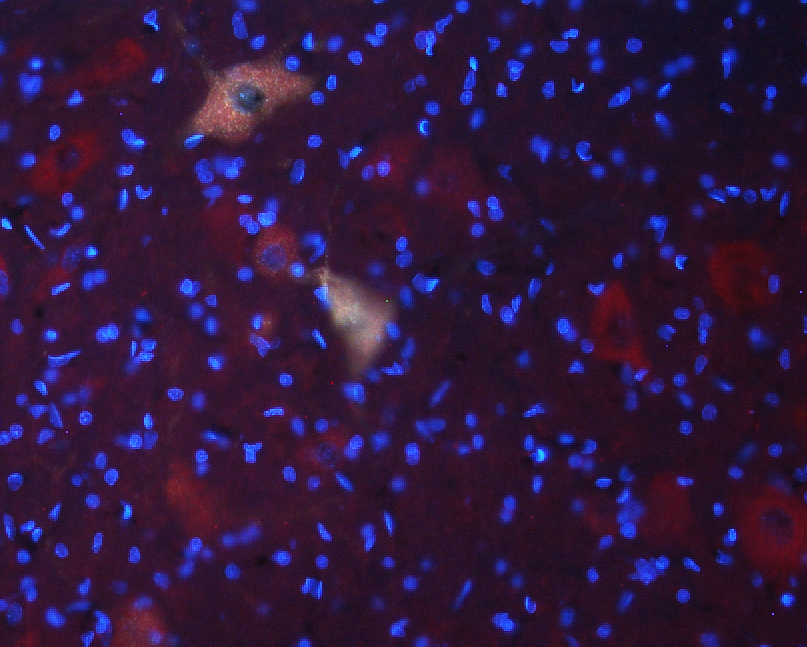

Supplement: Data S4 [file peerj-04-2025-s004.zip › raw data-figure 5/figure 5C/SCI-TT-BTX-merged.tif]

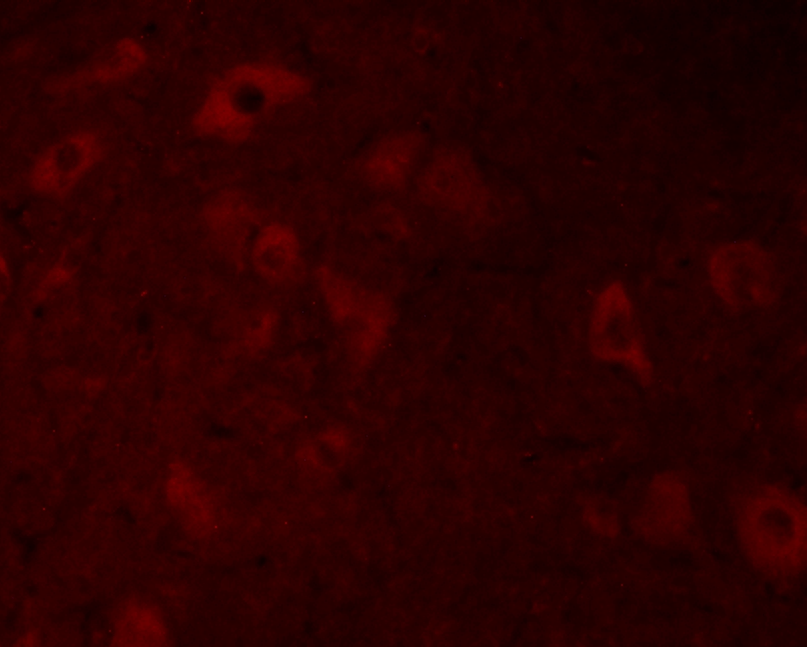

Supplement: Data S4 [file peerj-04-2025-s004.zip › raw data-figure 5/figure 5C/SCI-TT-BTX-TrkB.tif]

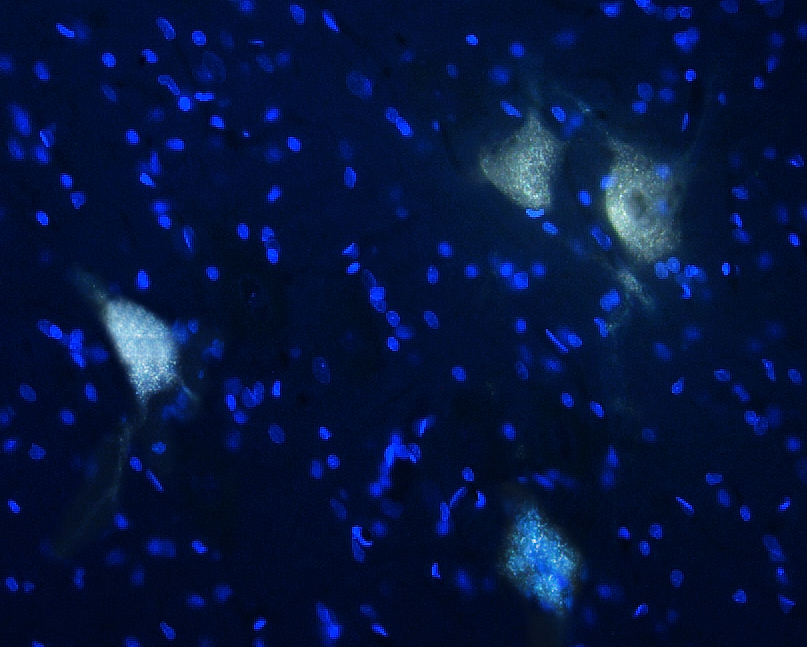

Supplement: Data S4 [file peerj-04-2025-s004.zip › raw data-figure 5/figure 5C/SCI-TT-Sal-FG.tif]

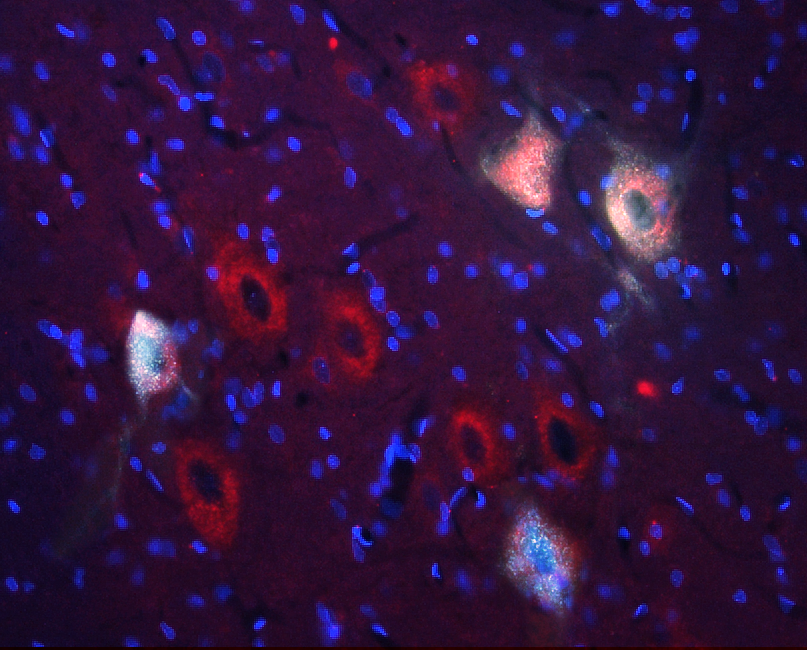

Supplement: Data S4 [file peerj-04-2025-s004.zip › raw data-figure 5/figure 5C/SCI-TT-Sal-merged.tif]

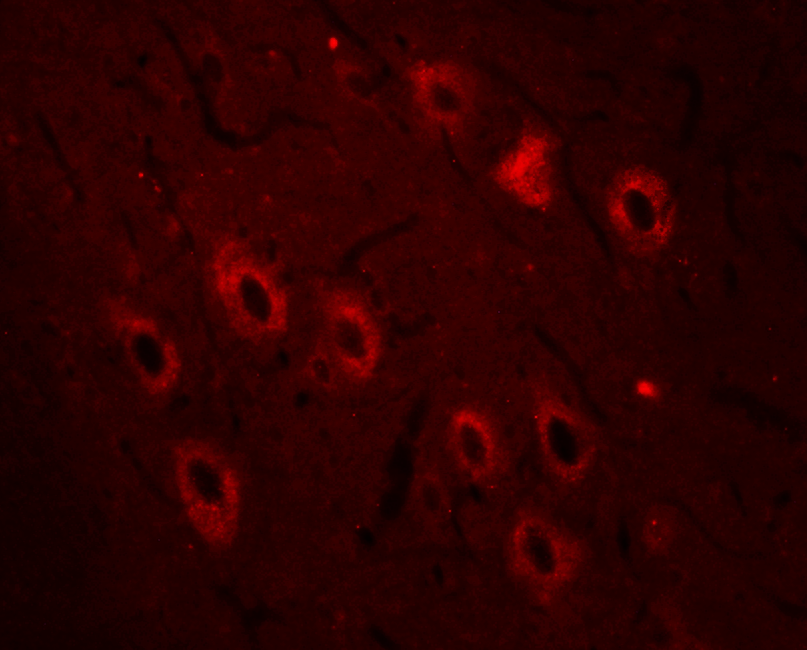

Supplement: Data S4 [file peerj-04-2025-s004.zip › raw data-figure 5/figure 5C/SCI-TT-Sal-TrkB.tif]

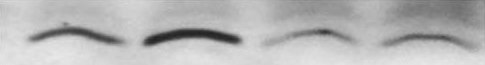

Supplement: Data S4 [file peerj-04-2025-s004.zip › raw data-figure 5/figure 5E/BDNF.jpg]

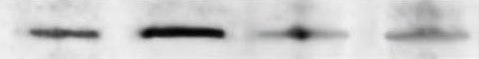

Supplement: Data S4 [file peerj-04-2025-s004.zip › raw data-figure 5/figure 5E/TrkB.jpg]

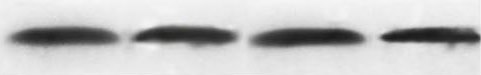

Supplement: Data S4 [file peerj-04-2025-s004.zip › raw data-figure 5/figure 5E/tubin.jpg]

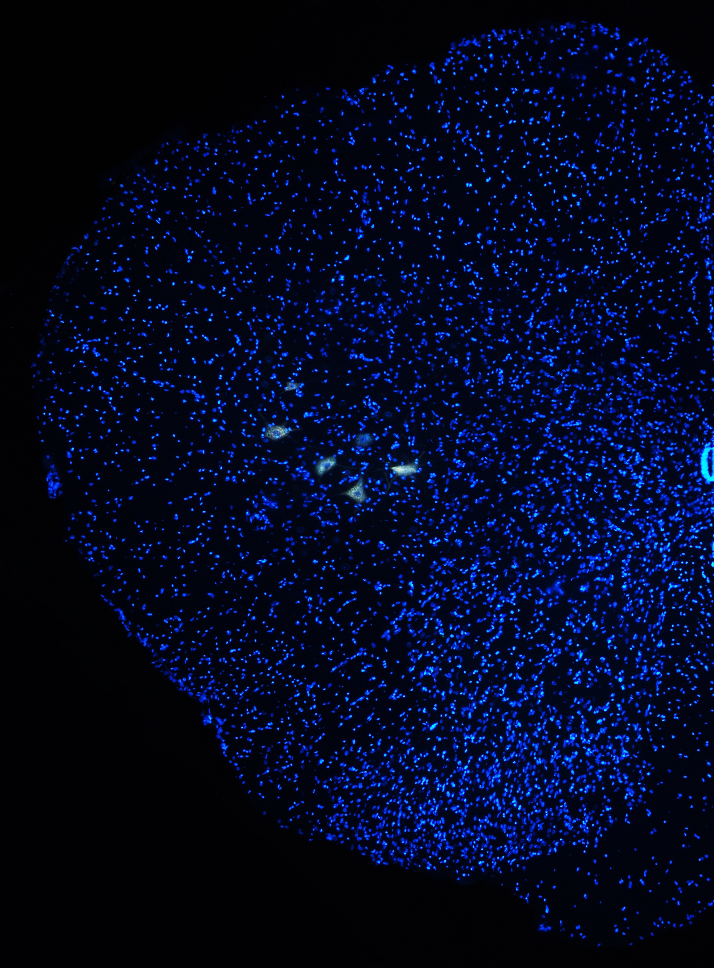

Supplement: Data S5 [file peerj-04-2025-s005.zip › raw data-figure 4/figure 4A/FG.tif]

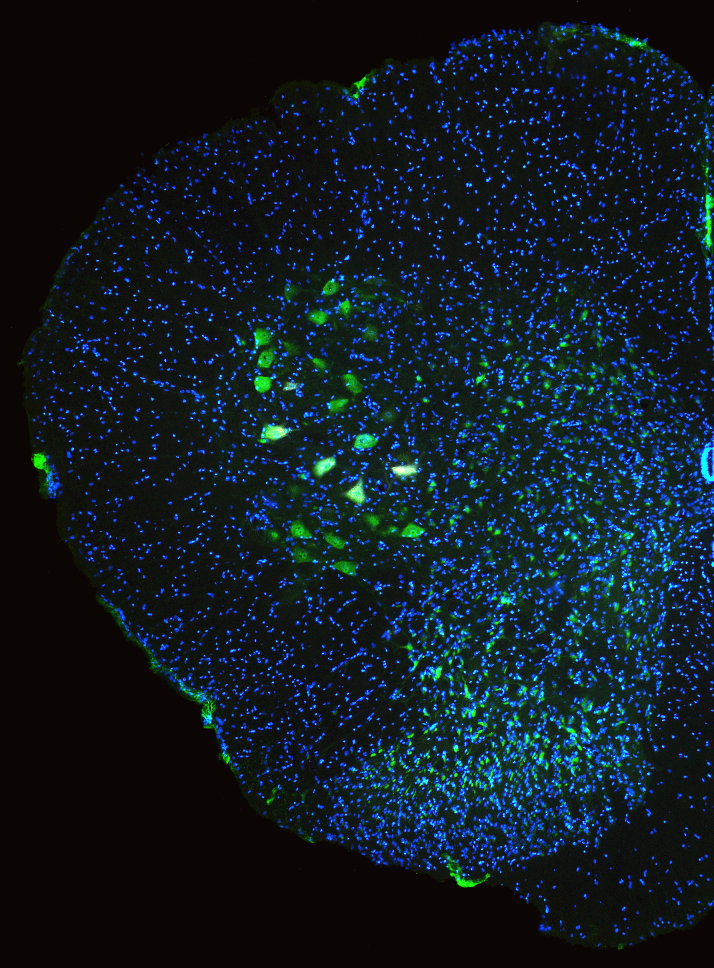

Supplement: Data S5 [file peerj-04-2025-s005.zip › raw data-figure 4/figure 4A/Merged.tif]

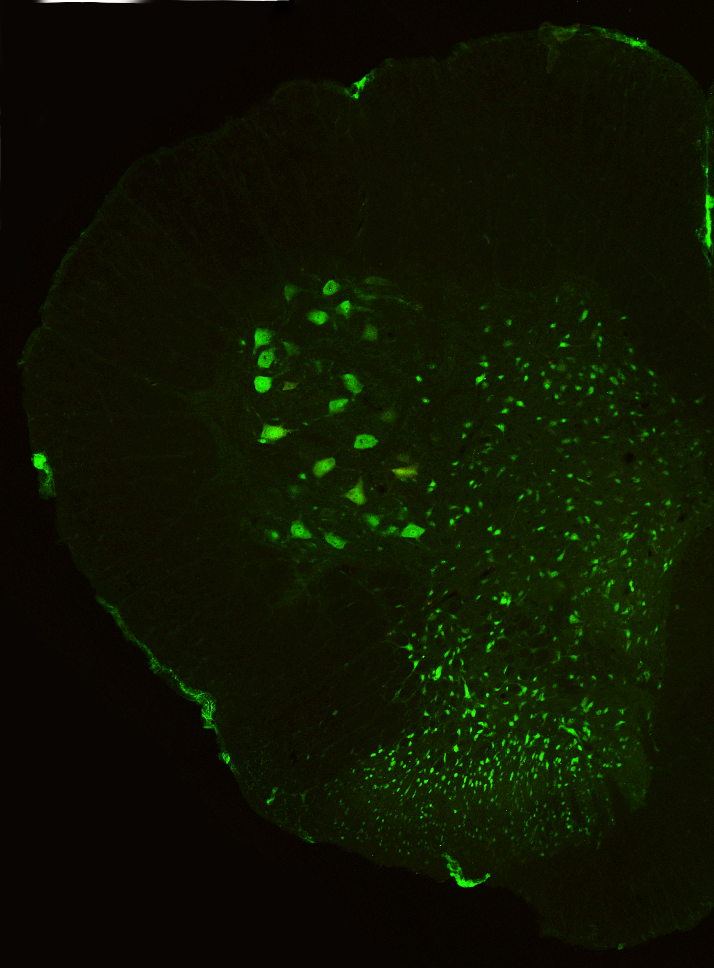

Supplement: Data S5 [file peerj-04-2025-s005.zip › raw data-figure 4/figure 4A/NeuN.tif]

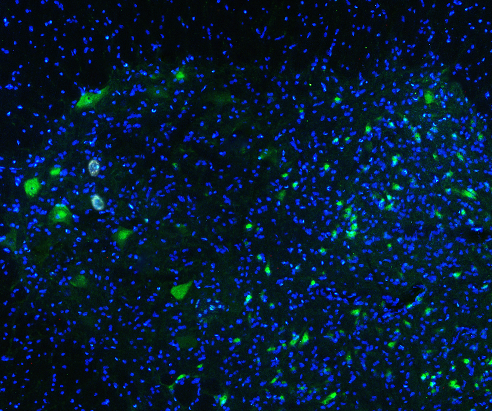

Supplement: Data S5 [file peerj-04-2025-s005.zip › raw data-figure 4/figure 4A/SCI-BTX.tif]

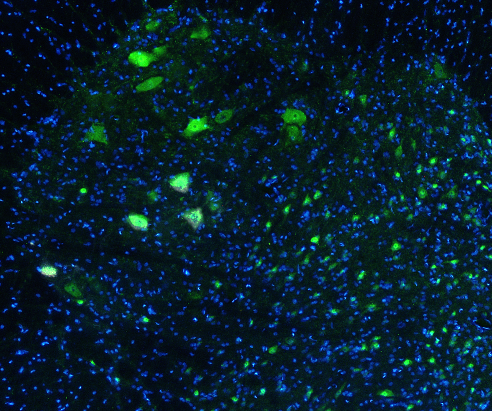

Supplement: Data S5 [file peerj-04-2025-s005.zip › raw data-figure 4/figure 4A/SCI-Sal.tif]

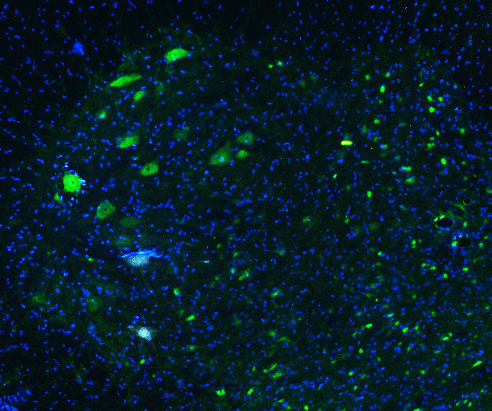

Supplement: Data S5 [file peerj-04-2025-s005.zip › raw data-figure 4/figure 4A/SCI-TT-BTX.tif]

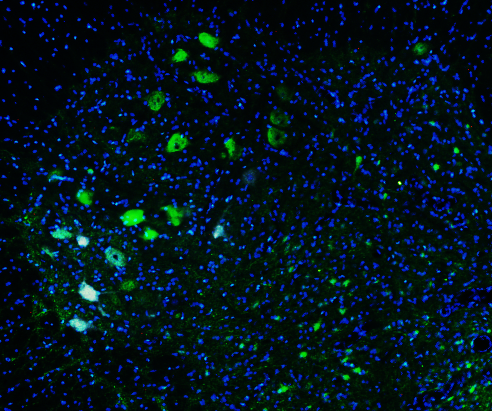

Supplement: Data S5 [file peerj-04-2025-s005.zip › raw data-figure 4/figure 4A/SCI-TT-Sal.tif]

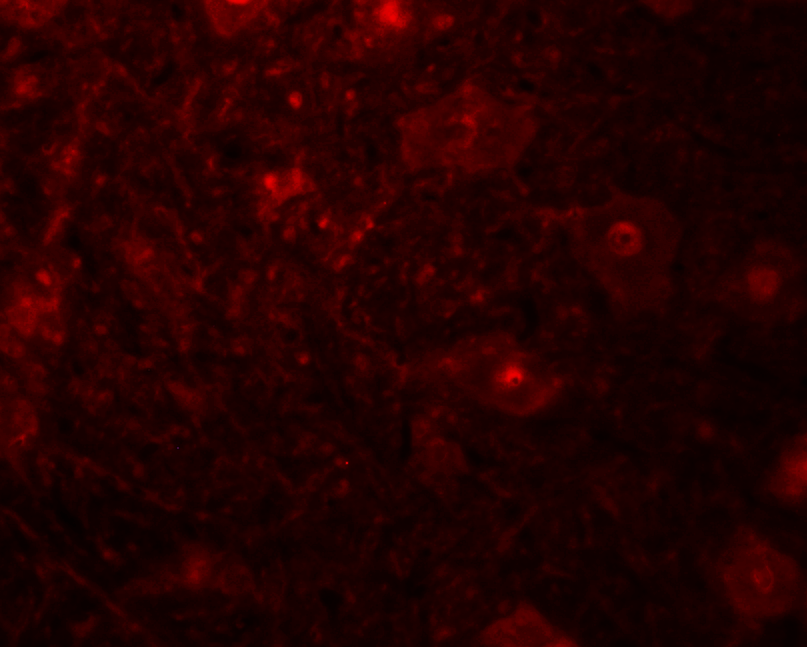

Supplement: Data S5 [file peerj-04-2025-s005.zip › raw data-figure 4/figure 4C/SCI Sal-c-fos.tif]

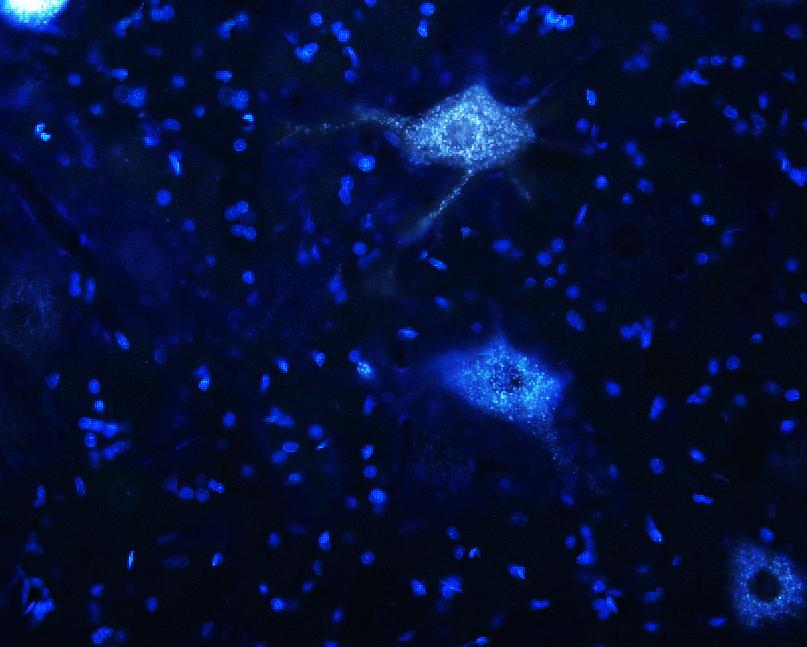

Supplement: Data S5 [file peerj-04-2025-s005.zip › raw data-figure 4/figure 4C/SCI Sal-FG.tif]

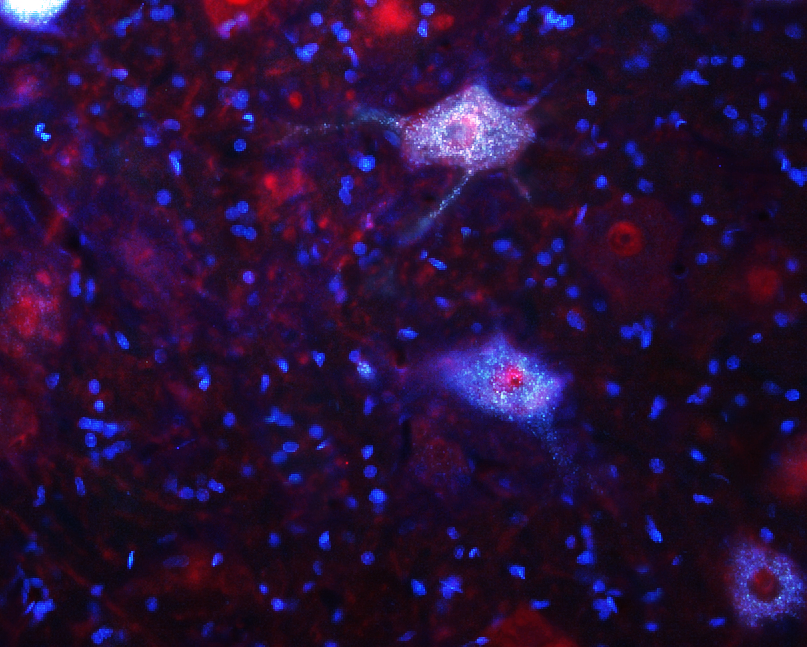

Supplement: Data S5 [file peerj-04-2025-s005.zip › raw data-figure 4/figure 4C/SCI Sal-merged.tif]

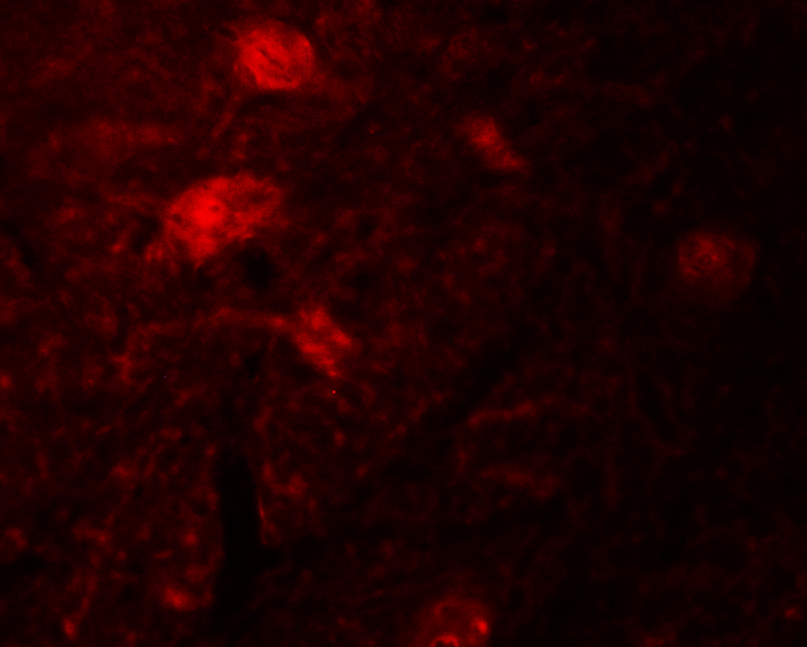

Supplement: Data S5 [file peerj-04-2025-s005.zip › raw data-figure 4/figure 4C/SCI-BTX-c-fos.tif]

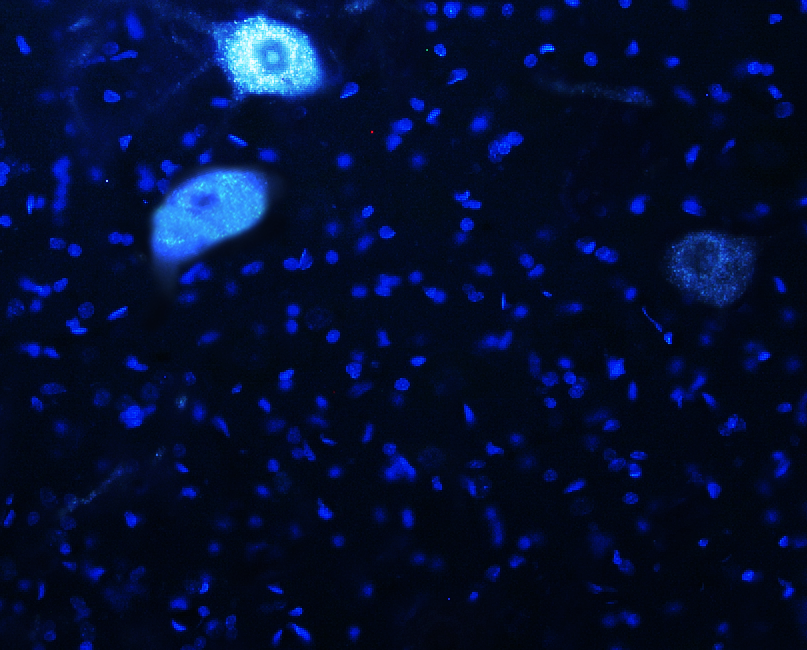

Supplement: Data S5 [file peerj-04-2025-s005.zip › raw data-figure 4/figure 4C/SCI-BTX-FG.tif]

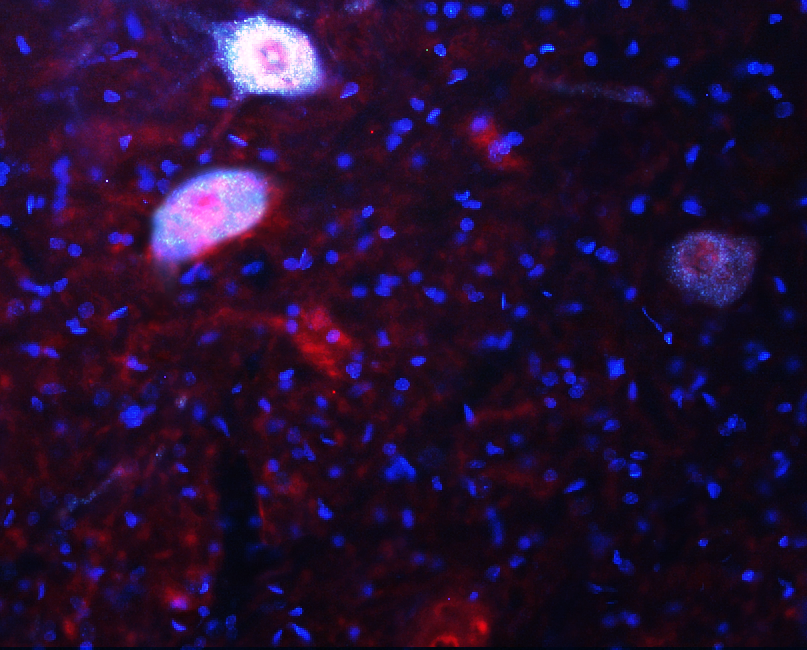

Supplement: Data S5 [file peerj-04-2025-s005.zip › raw data-figure 4/figure 4C/SCI-BTX-merged.tif]

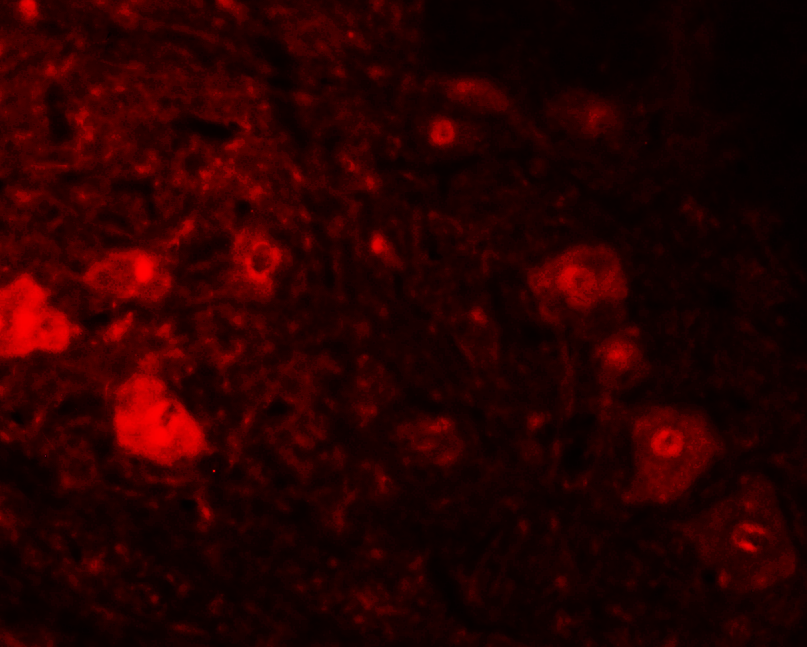

Supplement: Data S5 [file peerj-04-2025-s005.zip › raw data-figure 4/figure 4C/SCI-TT-BTX-c-fos.tif]

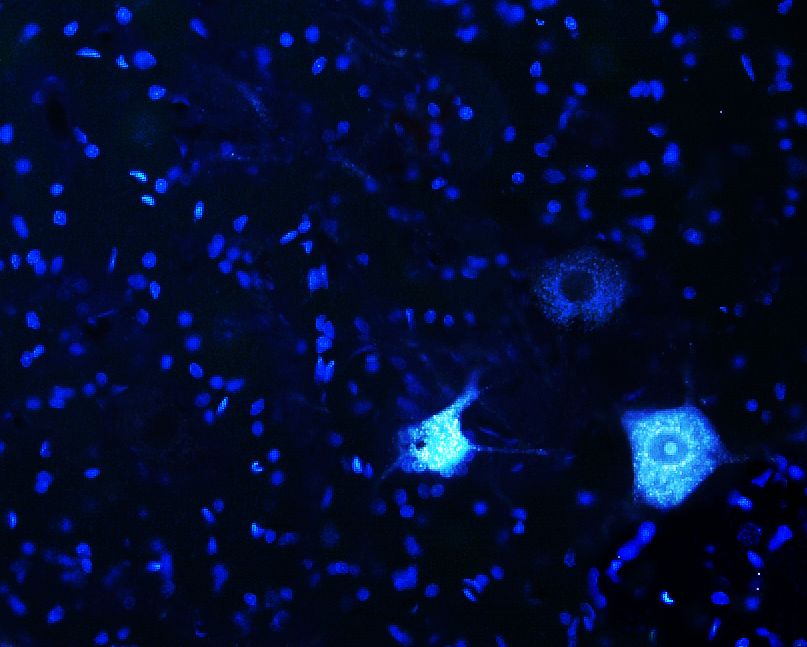

Supplement: Data S5 [file peerj-04-2025-s005.zip › raw data-figure 4/figure 4C/SCI-TT-BTX-FG.tif]

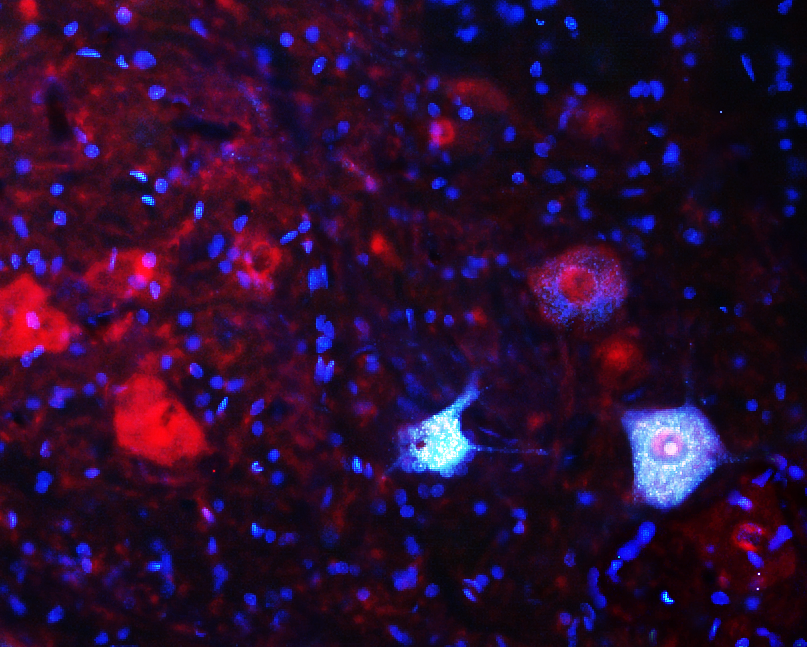

Supplement: Data S5 [file peerj-04-2025-s005.zip › raw data-figure 4/figure 4C/SCI-TT-BTX-merged.tif]

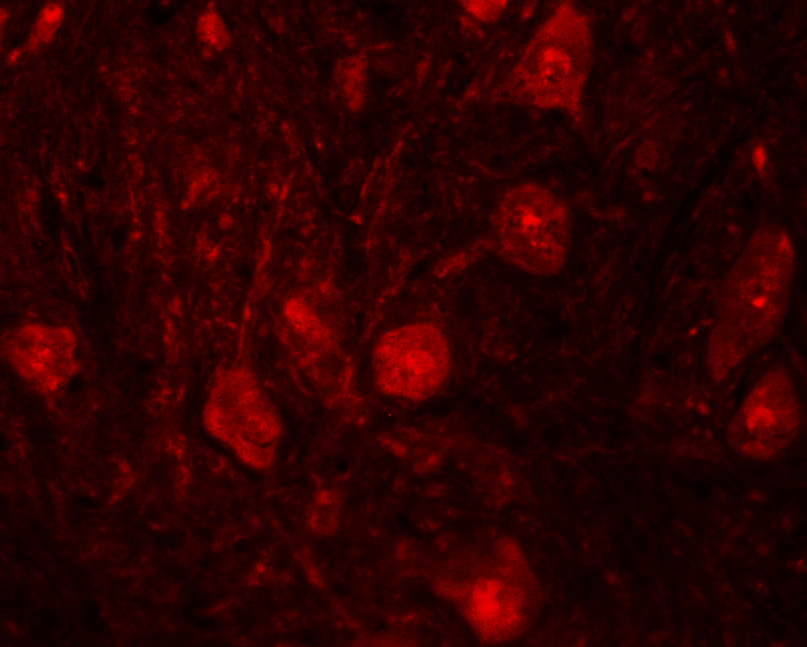

Supplement: Data S5 [file peerj-04-2025-s005.zip › raw data-figure 4/figure 4C/SCI-TT-Sal-c-fos.tif]

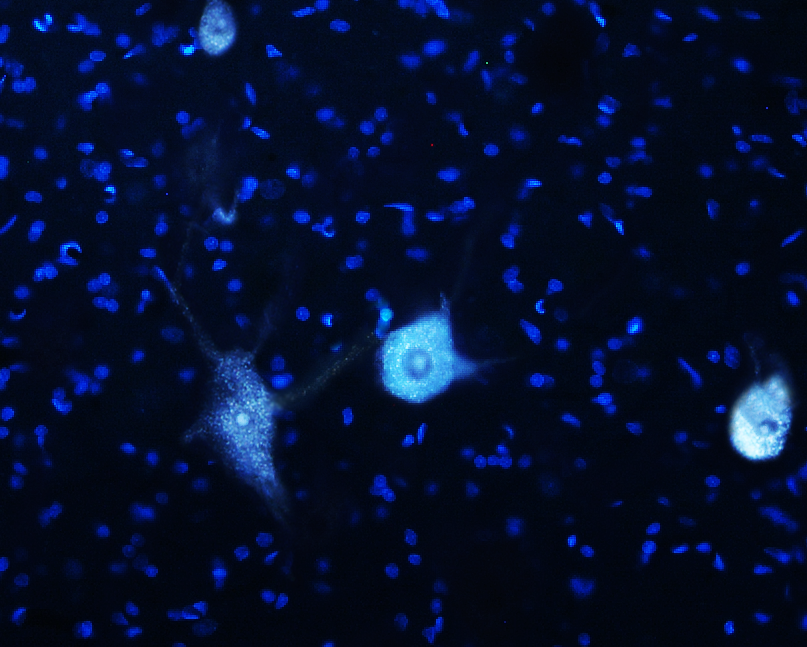

Supplement: Data S5 [file peerj-04-2025-s005.zip › raw data-figure 4/figure 4C/SCI-TT-Sal-FG.tif]

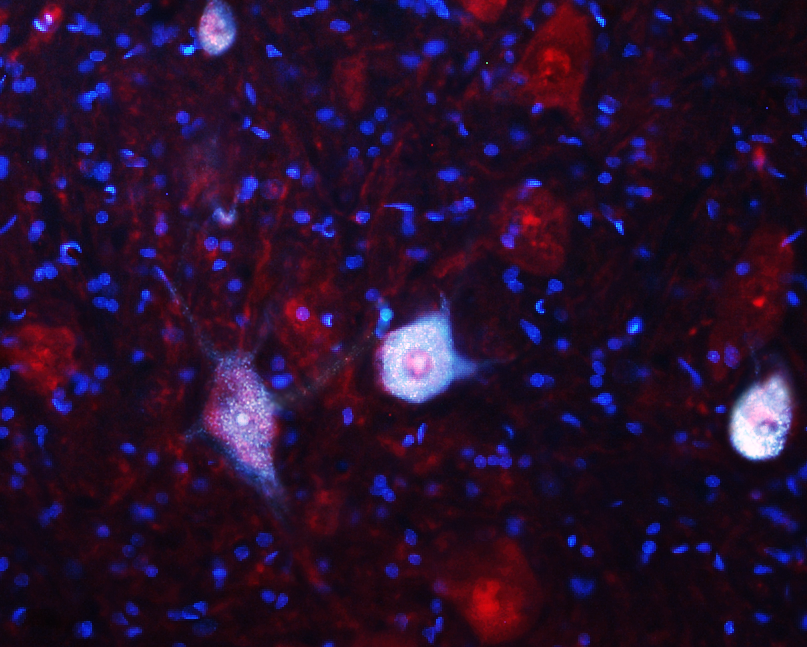

Supplement: Data S5 [file peerj-04-2025-s005.zip › raw data-figure 4/figure 4C/SCI-TT-Sal-merged.tif]

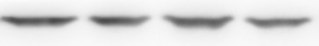

Supplement: Data S5 [file peerj-04-2025-s005.zip › raw data-figure 4/figure 4E/actin.tif]

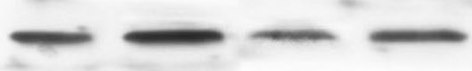

Supplement: Data S5 [file peerj-04-2025-s005.zip › raw data-figure 4/figure 4E/c-fos.jpg]

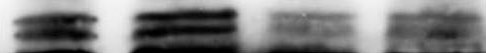

Supplement: Data S5 [file peerj-04-2025-s005.zip › raw data-figure 4/figure 4E/NeuN.jpg]
